# Methods for identifying adverse drug reactions in primary care: A systematic review

**DOI:** 10.1371/journal.pone.0317660

**Published:** 2025-02-04

**Authors:** Vera Logan, David Hughes, Adam Turner, Neil Carter, Sue Jordan

**Affiliations:** 1 Department of Nursing, Faculty of Health and Social Studies, University of South Bohemia, Ceske Budejovice, Czechia; 2 Faculty of Medicine, Health and Life Sciences, Swansea University, Swansea, United Kingdom; University of Oxford, UNITED KINGDOM OF GREAT BRITAIN AND NORTHERN IRELAND

## Abstract

**Background:**

Identification of real-time adverse drug reactions [ADRs] (as opposed to the risk of ADRs) in older poly-medicated people in primary care is a challenging task, often undertaken without an explicit strategy. This systematic review aims to evaluate replicable instruments and methods for identifying and addressing ADRs.

**Methods:**

A systematic search was conducted in Medline, CINAHL, Scopus, Web of Science and Cochrane library, using controlled vocabulary (MeSH) and free-text terms. Randomised controlled trials (RCTs) implementing strategies to identify or resolve ADRs experienced by patients in primary care were included. Two reviewers independently screened studies, extracted data, and assessed the risk of bias using the Cochrane Risk of Bias tool. Discrepancies were resolved by discussion.

**Results:**

From 2,182 unique records, 49 studies were identified for full review. Eight papers reporting results from 6 RCTs were included. All six trials utilised a list of medicine-related unwanted symptoms to identify ADRs. Two of three studies using adverse drug reaction questionnaires reported statistically significant increased rates of ADR reporting. Two of three studies that combined symptom questionnaires with prescriber consultations reported reductions in the number of health problems. Overall, results suggest that the three studies that described multidisciplinary collaborations using lists of ADRs plus prescriber reviews enhanced patient safety. However, the RCTs were unblinded and reported suboptimal retention. When considered as a whole, findings are equivocal and the data are too heterogenous to warrant any firm conclusions, beyond the need for more research to optimise strategies to safeguard patient wellbeing.

**Implications:**

Adaptable and scalable instruments with decision support are needed in primary care to identify and mitigate medicine-related harm in older poly-medicated people. The effectiveness of adverse drug reaction identification instruments, the value of comprehensive instruments, and the optimum method of delivery should be explored in multicentre trials.

## Introduction

Adverse drug reactions (ADRs) are persistent, leading causes of iatrogenic injury, avoidable harm and financial costs to healthcare providers and society [1]. ADRs are defined as ‘appreciably harmful or unpleasant reactions, resulting from an intervention related to the use of a medicinal product, which predict hazard from future administration and warrant prevention or specific treatment, or alteration of the dosage regimen, or withdrawal of the product’ [2], p. 9237]. Adverse drug events (ADEs) are a portmanteau term encompassing ADRs, subtherapeutic effects of therapy, drug dependence, intoxications and untreated indications [3]. ADRs affect one in six hospitalised patients globally [4], and 85% of older patients with dementia [5]. One in 12 unplanned hospital admissions are related to ADRs [6]. The NHS costs of ADRs attributable to error are estimated at £98.5 million per year [7], and non-indicated and unnecessary prescribing of dependency-forming medicines costs England’s NHS ~ £500,000 each year [8].

In the context of rising workload pressures in primary care, decreasing continuity of care, and the increasingly transactional nature of general medical practitioner (GP)/service user interactions [9,10], the questions of how primary care prescribers ascertain whether their prescribing leads to ADRs, and with what effect, are well-founded. Voluntary reporting and structured medication reviews are the main sponsored strategies to identify ADRs; however, they have their limitations, namely under-reporting [11], and considerable variation in implementation, detracting from their effectiveness [12].

The formulaic prediction of ADRs in older people may be problematic. Such patients are often frail, poly-medicated, with multiple co-morbidities and may receive long-term, repeat prescriptions for potent, high-risk medicines without individualised dose titration. Polypharmacy or poly-medication, in the sense of individuals being prescribed 5 or more regular medicines [13,14], increases the potential for drug-drug interactions and ADRs [15], medication errors [16], drug-related hospital admissions [17,18]. Poly-medication disproportionately affects the old and the poor [19,20]. Medicine-related harm is viewed as a wider term encompassing ADRs but also including errors in prescribing, dispensing and administering medicines [1]; errors are associated with polypharmacy and patient harm [16], and amenable to ADR monitoring interventions [21].

Detection of ADRs may be complex and highly individualised. Adverse reactions and medicine-related harm, such as dizziness, emesis, or cardiac arrhythmias, could be prevented with improved real-time monitoring for ADRs [22], in addition to ADR risk detection and rationalisation of prescribing [23]. A scripted, replicable trigger instrument that could be used with minimal training by a range of health professionals could contribute to medication safety [24,25]. However, there is a gap in the current literature regarding the impact of ADR monitoring approaches on identification and reduction of ADRs in all patients.

Three recent systematic reviews on ADR identification illustrate the importance of the topic, yet none assessed the effects of real-time ADR identification instruments, rather than identification of potential ADRs from retrospective reviews of patients’ notes. One review [26] identified such instruments, whilst the other two [12,27] focussed on prescribing, see Table 1.

**Table 1:** Conclusions from other systematic reviews.

Review first author and year	Review aims	Review results and conclusions
**Lim, 2022 [** **26** **]**	To identify published patient-reported ADR questionnaires and to summarise the psychometric properties of the questionnaires.	19 patient-reported ADR questionnaires were identified. Around 58% of the questionnaires (11 studies) assessed content validity, 80% had patient input during development, 42% (8 studies) were tested for internal consistency, and 37% (7 studies) for test-retest reliability. More robust validity and reliability testing is needed when developing patient-reported ADR questionnaires.
**Atmaja, 2022 [** **27** **]**	To identify and evaluate reports on tools that predict and detect ADR in elderly patients ( ≥60 years).	ADR prediction tools (such as the STOPP/START version 1 and 2) did not reduce the number of drug interactions within 2 months (OR 0.84 [0.70–1.02], P 0.08, two studies) and mortality (OR 0.92 [0.76–1.12], p = 0.41, three studies). No definitive and validated assessment tool for detecting a predicting ADR in elderly patients was found.
**Gray, 2023 [** **12** **]**	To update a previous systematic review reporting the impact of interventions to optimize medication use on ADRs in older adults.	6 new studies were added to a previous review by the same group, resulting in a total of 19 studies. Interventions were mostly pharmacist-led medicine reviews including implicit methods of ADR identification (10 studies). The intervention group participants were 19% less likely to experience an ADR (OR 0.81, 95%CI 0.48–0.96) and 32% less likely to experience a serious ADR (OR 0.68, 95%CI 0.48–0.96). Pharmacist-led interventions appeared more effective at reducing the risk of any ADR, compared with other types of intervention (other healthcare professional-led, technology-based or educational), but this was only marginally statistically significant. Interventions, particularly when involving pharmacists, significantly and substantially reduced the risk of ADRs and serious ADRs in older adults, but stronger evidence is needed.

To fill the knowledge gap, this systematic review aims to:

1) Provide a comprehensive overview of (recognised) current methods of real-time ADR identification, suitable for use by a variety of health professionals with minimal training in medicines management in community care.
2) Establish their effects in identifying and ameliorating ADRs.

This review is reported in accordance with the Preferred Reporting Items for Systematic reviews and Meta-Analyses (PRISMA) 2020 schema [28].

The protocol for this search is registered in the international prospective register of systematic reviews (Prospero) [29]: https://www.crd.york.ac.uk/PROSPERO/display_record.php?RecordID=322374 and the initial search strategy is available: https://www.crd.york.ac.uk/PROSPEROFILES/322374_STRATEGY_20220331.pdf [30].

## Methods

### Search strategy

Four bibliographic databases (Medline, CINAHL, Scopus, Web of Science) and the Cochrane Library were searched up until August 2024. Additional manual backward and forward reference searches were conducted to retrieve pertinent key publications by cross-referencing bibliographies. We searched for systematic reviews to check reference lists for any papers not identified by our search strategy.

The three foci of the search were: ADRs, detection instruments and primary care. A preliminary PubMed/Medline test of the search terms was undertaken to identify the major terms and provide a rationale for adjusting the search strategy. Primary care setting terms were checked against the WHO glossary of terms [31]. The final search strategy was constructed using keywords, MeSH terms, and truncations. The search was confined to papers published in English. There were no temporal restrictions, other than the search end date (see S1 File for a full search strategy and results).

In line with the review aims, the eligibility criteria were as described in Table 2.

**Table 2:** Selection criteria.

	Inclusion	Exclusion
**Study design**	Randomised controlled trials of all types	Phase IV post-marketing surveillance studies
**Setting**	Primary care Service users living in their own homes, with or without support of family or services	Institutions delivering long-term care (including nursing homes) Specialist services (for example, substance misuse services, mental health, cancer or end of life care)
**Participants/population**	Adults (aged over 18) who take prescribed medicines in primary care	
**Intervention**	Studies describing the use of a method of identifying or identifying and resolving potential or actual ADRs in individual patients. The interventions could form part of wider, complex interventions. Interventions which: Were described in enough detail to be reproducible Could be administered to service users by a range of health professionals without specialist pharmacist training or self-administered by service users.	Pharmacovigilance systems contributing to pharmacoepidemiology and regulatory/drug safety authority decisions and therapy guidelines. Studies focusing on: Treatment adherence Blood/plasma/serum concentrations of specific medicines Pharmacokinetic studies Effects of off-label and unlicenced prescribing Substance misuse diagnostic instruments
**Medicines**	General medicines, typically prescribed in primary care for long-term conditions A range of medicines A broad group of medicines, for example, mental health medicines.	Single specific medicines (for example, warfarin) Single narrow specialist medicine group (for example, anticoagulants or opioids) Medicines for a single condition that is relatively uncommon in the UK (for example, tuberculosis) Medicines not related to chronic conditions treatment (for example, antibiotics) Safety profiles of specific medicines Effectiveness of specific medicines

### Study selection

A three-stage screening process was used to identify studies meeting the inclusion criteria. At stage one, one author (VL) screened titles of studies and excluded work that clearly did not fit the inclusion criteria, based on the title. Where the first reviewer was not certain about study eligibility, the study was kept for further assessment at stage two. A random sample of fifty titles was independently screened by a second reviewer (SJ).

Stage two included independent title and abstract screening by two reviewers (VL and SJ or VL and AT). The decision-making criteria are described in S1 Fig. Any disagreements between two reviewers were resolved by a third reviewer. At stage three, full texts of the shortlisted papers were thoroughly checked to ensure they met the requirements in terms of relevance and quality by the author and independently by a second reviewer (SJ).

### Data extraction

Data extraction was informed by PRISMA guidelines [28] (S2 Fig). The *ad hoc* data collection form elements included: reference, country, dates of study, aims, design, sample and setting, ADR identification intervention, outcome measures, results, and conclusions. Data were compiled for the following outcomes and data variables: range of intervention-relevant medicines, type of population the intervention targeted, effects of the instrument in identifying ADRs (number and nature of ADRs), intervention nature and complexity, clinical outcomes, and implementation evaluation.

### Assessment of study quality

Two authors (SJ, VL) independently assessed the methodological quality of the extracted studies, utilising the Cochrane Risk of Bias tool (RoB2) [32], or Cochrane Risk of Bias for cluster-randomised trials (RoB2CRT) [33]. Disagreements over the risk of bias in given studies were resolved by discussion and arbitration by a third reviewer (AT). Certainty in the body of evidence was assessed using the Grading of Recommendations, Assessment, Development and Evaluation (GRADE) approach [34,35].

### Outcomes

We sought to identify the number and types of ADRs identified, changes in processes and outcomes of care, and benefits and disbenefits associated with each intervention, as reported by trialists.

### Analysis

The study intervention characteristics were extracted and tabulated to describe and compare the findings. A form was created for each included study, in a brief, logical and descriptive format where information was organised under pre-specified headings. The forms were subsequently analysed and compared, and the tabulated findings formed the baseline for summarising and integrating the findings. Due to the variety of study designs, measured outcomes, and the small number of trials, studies’ findings were narrated without statistical synthesis and meta-analysis, as these were too heterogeneous to yield meaningful results [36].

## Results

The searches yielded 2,182 titles, comprising 381 from Medline, 185 from CINAHL, 820 from Scopus and 796 from Web of Science. 497 duplicates were removed, and 1264 articles were excluded based on the title not fitting inclusion criteria, leaving 421 studies for further evaluation. No disagreements were identified in the random sample check: of the 50 double-reviewed titles, both reviewers (VL/SJ) selected the same 6 for inclusion, and 44 for exclusion. Details of studies retained at stage 1 can be found in S2 and S3 Files.

**Fig 1:**
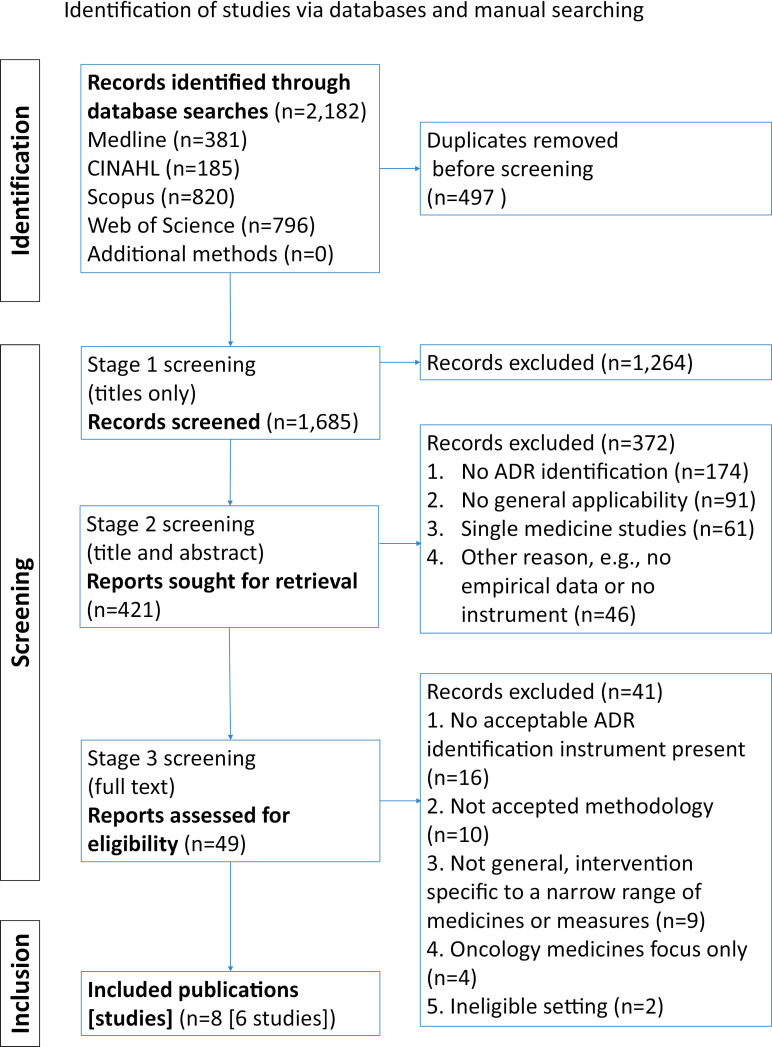
PRISMA flow diagram.

372 studies were removed at stage 2 screening: 174 did not identify ADRs in the study (‘no ADR identification’); 91 studies considered only one or two specific ADRs or abnormalities in laboratory tests results (‘no general applicability’); 61 studies examined only a single medicine or a narrow medicine range (‘single medicine studies’); 46 were excluded for ‘other’ reasons, such as ineligible setting or design of study (Fig 1). Details of studies excluded at stage 2 are reported in S1 Fig and S1 Table. Study exclusion was reported based on the first reason that became obvious, even though in some cases studies could have been excluded for multiple reasons.

41 further records were excluded after reading full texts at stage 3, which involved assessment of compatibility of ADR identification instruments with the inclusion criteria (Table 2). Most ineligible interventions lacked a replicable ADR identification instrument described in sufficient detail (n = 16), full details of excluded categories and studies are included in S2 Table. At stage 3, there was a high level of agreement between the two raters (95%), and only two articles were escalated for a third opinion. Removal of duplicates, combined with the 3-stage study screening selection process (Fig 1) led to identification of 8 relevant papers, relating to 6 RCTs (presented in this review as 6 studies).

### Characteristics of included studies

The eight papers extracted had publication dates between 2001 and 2021. Four of the identified papers [37–40] reported results from two trials, and for the purposes of this review were analysed as two studies (1 per trial). All six studies reported in the eight papers were randomised controlled trials. Two RCTs were cluster-randomised: one was cluster-randomised, with primary care clinics treated as the clusters [41]; and the other was a cluster-randomised, controlled cross-sectional stepped wedge open trial, where primary care medical (GP) practices represented the clusters [42]. Three RCTs randomised individual patients [39,43,44] and one study was block-randomised per pharmacy [37]. Three of the studies were conducted in the USA, and one each in Australia, France and the Netherlands. Key study information is included in Tables 3 and 4, and the interventions are described in detail in S4 File. Two trials monitored ADRs as components of complex interventions [37,43], and are tabulated separately (Table 4).

**Table 3:** Table of included studies – ADR identification is the main outcome of the study.

First author and year, country	Aims of study	Study design, sample size, setting	ADR^b^ identification intervention	Outcome measures	Results	Authors’ conclusions
**Weingard 2013, USA [** **44** **]**	To evaluate GP-MedCheck^a^ (automated electronic message system within patient internet portal) for enhancing communication about medication-related symptoms.	RCT 738 participants (375 intervention, 363 controls).	GP-MedCheck^a^ message asked patients issued a new prescription if they received the medicine and if they experienced any problems with the medicine, using a generic list of ADEs^b^.	Numbers of: ADEs^b^, preventable ADEs^b^, ameliorable ADEs^b^. Healthcare utilisation (number of telephone contacts, specialist appointments, emergency department or GP visits, admissions to hospital).	184 of 375 (49%) patients responded to MedCheck message. 52 unfilled prescriptions and 56 medication problems. There was no statistically significant difference between arms in the rate of ADEs or in healthcare utilisation.	Internet portals have the potential to enhance patient- physician communication. Additional development is required to demonstrate that such interventions can improve medication safety or health-care utilization.
**Schoenmakers 2017 and 2018, Netherlands [39**,**40]**	(a) To compare changes in patient-reported ADRs^c^ collected by PROMISE^d^ before and after community pharmacist-led clinical medication reviews with usual care. (b) To describe numbers and types of patient-reported symptoms assessed during clinical medication review and their association with medicines use.	RCT 228 consented, 180 completed first data collection, 145 participants completed second data collection (78 intervention + 67 control participants), total of 83 lost to follow-up. Setting: community pharmacies.	PROMISE^d^ – Patient-Reported Outcome Measure, inquiry into side effects (22 symptom categories).	A - Mean number of drug-associated symptoms at follow-up. B - The number of patients reporting a pre-defined symptom. C - The number of patients reporting this as a drug-associated symptom and D - the number of patients reporting a symptom that was a ‘very common’ side effect in at least one of their drugs in the relevant SPC^e^.	A - Measures effect of pharmacist intervention, not PROMISE instrument. B - 168 of 180 patients (93.3%) reported at least one symptom, total of 1102 symptoms were reported in 22 symptom categories. C – 71.9% of symptoms were reported as definitely or possibly drug-associated. D – 107 of 180 patients (59.4%) reported 284 symptoms mentioned in the SPC as being ‘very common’ side effects of 65 medicines used.	PROMISE provided meaningful information on drug-associated symptoms in clinical medication reviews. However the number of drug-associated symptoms was not reduced by clinical medication reviews compared with usual care.
**Schiff 2018, USA [** **41** **]**	To evaluate an automated telephone surveillance system with transfer to a live pharmacist to screen potentially drug-related symptoms after newly starting medications for four common primary care conditions (hypertension, diabetes, depression and insomnia).	Cluster-RCT 776 instrument arm adult participants, 776 matched controls, newly prescribed one of the target medicines. Setting: 26 clinics (13 randomised to intervention).	Interactive voice response automated calling system (its script lists several common ADRs^c^).	Primary outcome: physician documentation of any adverse effects associated with the target newly-prescribed medicine.	320 of 776 participants were transferred to pharmacist with 1021 potential ADRs^c^. Compared to propensity-matched controls, intervention participants were significantly more likely to have ADRs documented in the medical notes (277 vs 164 ADRs^c^, p < 0.0001).	The automated calling system was effective at detecting the ADEs^b^, but presents challenges, such as high loss to follow-up, likely due to people being tired of automated telephone calls.
**Buchet-Poyau 2021, France [** **42** **]**	To assess the impact of the educational booklet intervention on patient self-reporting to GP any ADEs^b^ relating to antihypertensives.	Cross-sectional stepped wedge cluster-randomised trial, 60 GPs, 8 clusters, 1102 patient participants, 1095 analysed (546 instrument and 549 control).	Impact programme – Educational booklet (information on cardiovascular risks, management of treatment, and ADEs^b^) to facilitate ADR^c^ reporting.	Primary: one or more patient self-reported ADEs^a^. Secondary: one or more patient or GP- reported ADEs^b^.	Primary outcome: Control: 2.4% (13/549). Instrument: 5.7% (31/546). Secondary outcome: Control: 9.3% (51/549). Instrument: 12.3% (67/549).	A booklet can improve patient self-reporting of ADEs to GPs. Future research should assess whether it can improve general practitioner management of ADEs and patients’ health status.

Studies ordered chronologically.

^a^GP-MedCheck – an automated electronic message generated in a patient Internet portal.

^b^ADE – adverse drug event.

^c^ADR – adverse drug reaction.

^d^PROMISE – Patient-Reported Outcome Measure, Inquiry into Side Effects.

^e^SPC – Summary of Product Characteristics.

**Table 4:** Table of included studies – ADR identification is part of a wider intervention.

First author and year, country	Aims of study	Study design, sample size, setting	ADR^a,b^ identification intervention	Outcome measures	Results	Authors’ conclusions
**Jameson 2001, USA [** **43** **]**	To investigate cost and adverse effect outcomes associated with a pharmacotherapy consultation.	RCT 340 – 72 lost to follow-up or protocol violations. 268 analysed (144 control, 124 instrument arm) participants with 5 or more prescriptions. Ambulatory care (4 large physician practice groups, 133 physicians consented).	Consultation – 45- to −60-minute interview (face to face). 18-item ADR^c^ questionnaire to assess ADRs^c,d^.	Changes in drug costs, medical costs, drug-related symptoms.	Intervention arm: 67 (54%) reduced symptoms of ADRs, 27 (22%) worsened ADR^c^ symptoms. Control arm: 58 (40%) reduced, 46 (32%) worsened. Only 27% of the actions needed were identified by chart review alone. The remaining 73% required an interview for the problem to be recognised.	73% of the original problems were recognized only through a patient interview. An interpersonal relationship remains critical to the provision of pharmaceutical care. Broad-based interventions in complicated patients are too difficult to evaluate accurately.
**Verdoorn 2019 a and b [37**,**38] Netherlands**	To investigate whether goal attainment scaling (GAS) is a useful tool for determining goals and monitoring their attainment during clinical medication review.	A - Pragmatic RCT. B - Subset analysis of the intervention group of the larger pragmatic RCT. 2290 invited, 707 consented, 629 randomised participants (315 intervention participants, 314 control). 41 left trial early, 588 completed (294 intervention, 294 control).	Person-centred clinical medication review, focused on personal goals, health-related quality of life and the number of health problems. Goal attainment scales used.	Goal attainment (e.g., to reduce pain or dizziness).	406 health-related goals were set for 283 of 315 included persons (90%). Implementation rate of GAS-related DRPs^e,f^ was 81%, compared with 62% for non-GAS-related DRPs^f^.	Including the patient’s personal goals and preferences in a medication review may help to establish the effects of increasing quality of life measured with EQ-VAS^g^ and decreasing the number of health problems, which are outcomes that are relevant to older patients’ lives.

Studies ordered chronologically.

^a^ADE – Adverse drug event.

^b^ADR – Adverse drug reaction.

^c^GP-MedCheck – An automated electronic message generated in a patient Internet portal.

^d^PROMISE – Patient-Reported Outcome Measure, Inquiry into Side Effects.

^e^SPC – Summary of Product Characteristics.

^f^DRP – Drug-Related Problems.

^g^EQ-VAS – European Quality of Life Visual Analoque Score.

The total aggregated sample in the six trials was 4,605 consenting participants. 178 participants (3.87%) were lost to follow-up or excluded due to protocol violation, resulting in a final sample of 4,427. Three studies [41,42,44] included adult participants (≥18 years of age) who had recently received a new prescription. One of them [42] also included participants whose prescriptions had been modified or renewed. The other three studies targeted poly-medicated patients, with two focusing on older patients. One study [39] recruited participants ≥65 years of age, while the second study [37] applied an inclusion threshold of ≥70 years of age. The third polypharmacy trial [43] set its inclusion criterion as age 6 and above, although the youngest participant recruited was over 40 years of age.

Four of the six studies did not specify the types of medicines that could be included. Three of the four [37,39,43] stipulated that medicines must be used long-term or for chronic conditions, while one [44] did not set any criteria. One study [41] targeted medicines for hypertension, diabetes, insomnia and depression, and one trial [42] included participants prescribed any antihypertensive medicines.

The statistical constructs used to compare outcome data between two groups varied between studies. Most studies used binary outcomes, with the exception of one [43], which used ordinal outcomes. Significant clinical, methodological, and statistical heterogeneity was observed between the studies (Table 5). The studies offered insufficient detail to describe clinical benefits and long-term *sequelae*.

**Table 5:** Heterogeneity in the included studies.

Study	Type of outcome	Effect measure
**Jameson and van Noord, 2001 [** **43** **]**	Ordinal	Number of patients with improving, same or worsening ADE symptoms.
**Weingard et al., 2013 [** **44** **]**	Binary	Number of ADEs, preventable ADEs and ameliorable ADEs.
**Schiff et al., 2018 [** **41** **]**	Binary	Physician documentation of any adverse effects.
**Schoenmakers et al., 2017 [39**,**40]**	Binary	Mean number of drug-associated symptoms at follow-up.
**Verdoorn et al., 2019 [37**,**38]**	Binary	Number of health problems after 3 and 6 months.
**Buchet-Poyau et al., 2021 [** **42** **]**	Binary	Reporting at least one ADE by patient to GP in 3 months post enrollment.

### Methodological quality of studies

**Fig 2:**
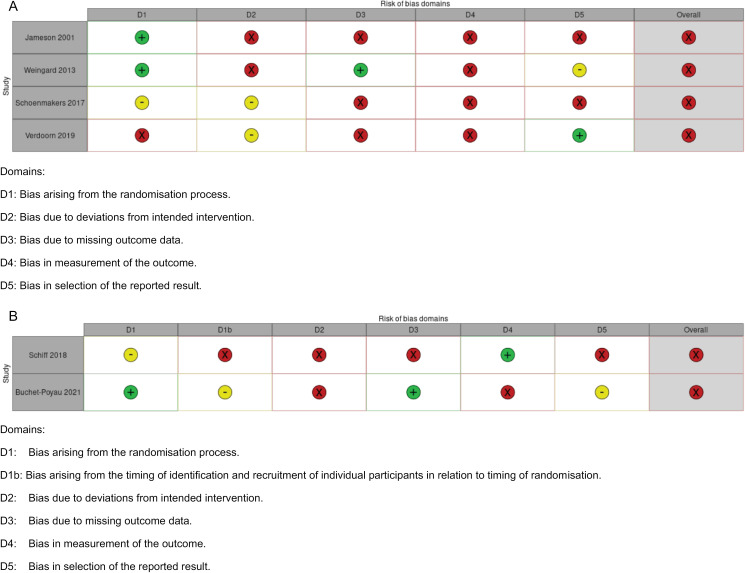
Risk of bias summary. (a) Individually randomised studies. (b) Cluster-randomised studies.

Cochrane Risk of Bias summary of the assessments is presented using the ‘robvis’ [45] visualisation tool (Fig 2a and 2b). All identified studies had a high overall risk of bias, reflecting issues within different aspects of trial design, conduct and reporting. All studies had a risk of bias due to the impossibility of blinding, where the participants and people delivering interventions were aware or likely aware of the intervention groups. This might have affected data assessment, to an unknown extent. Some studies were at risk of bias due to missing outcome data, where participants were lost to follow-up [37,43] or violated the protocol [43], did not complete the study, possibly due to sickness [39,41] or due to lack of relevant symptoms [44]. The true effect of the interventions may, therefore, be systematically underestimated or overestimated, with unpredictable overall direction.

### Results of individual studies

#### Differences in ADR identification methods.

At the core of each of the six methods of ADR identification in the studies reviewed was a list of medicine-related unwanted symptoms. The lists varied widely in the level of comprehensiveness, content, and function within the intervention and method of delivery. ADRs were assessed using between 12 and 25 items, with dizziness, rash, diarrhoea and fatigue represented in all lists, see Table 6.

**Table 6:** ADR symptom lists in the identified studies.

Symptom captured in ADRe	Jameson et al., 2001	Weingard et al., 2013	Schoenmakers et al., 2017	Schiff et al., 2019	Verdoorn et al., 2019
**Constipation**	✓	✓	✓	✓	✓
**Dizziness/light-headedness**	✓	✓	✓	✓	✓
**Rash, skin problems, itching**	✓	✓	✓	✓	✓
**Diarrhoea**	✓	✓	✓	✓	✓
**Mood changes, low mood**	✓	✓	✓	✓	
**Nausea/vomiting**	✓		✓	✓	
**Headache**	✓	✓	✓	✓	
**Fatigue, weakness**	✓		✓	✓	✓
**Sexual function problems**	✓	✓	✓	✓	
**Stomach problems**		✓	✓	✓	
**Dry mouth**	✓		✓		✓
**Bladder problems**		✓			✓
**Sleep problems**	✓	✓			
**Pain**	✓	✓			✓
**Shortness of breath, wheezing**				✓	✓
**Dyspepsia**				✓	✓
**Vision problems**	✓		✓		
**Abdominal pain**			✓	✓	
**Muscle/joint pain**			✓	✓	
**Confusion**	✓			✓	
**Chest pain**	✓				
**Swelling**	✓				
**Cold hands/feet**	✓				
**Change of appetite**			✓		
**Mouth complaints**			✓		
**Dyspepsia**			✓		
**Flatulence**			✓		
**Palpitations**			✓		
**Shivering/trembling**			✓		
**Muscular weakness**			✓		
**Drowsiness**			✓		
**Bruising/bleeding**			✓		
**Sweating**			✓		
**Nightmares/bad dreams**	✓				
**Memory problems**				✓	
**Weight problems (lost or gained)**			✓		
**If diabetes, have you had a value <60 since starting medication**				✓	
**New cough**				✓	
**Swelling of face/lips/tongue**				✓	
**Sleeping too much**	✓				
**Little pleasure in doing things**				✓	
**Depression**				✓	
**Mobility problems**					✓
**Sedation**					✓
**Cognition**					✓
**Other (not specified)**		✓	✓		
**Total**	18	12	25	21	13

NB: study 32 list is not available.

Service users self-completed the symptom lists in three studies [37,39,44]. Two studies used automated approaches: electronic messaging [44] and automated telephone calls [41], and one study used their ADR checklist as an information providing/educational tool, rather than a data collection tool, reversing the flow of information from the clinicians to the participants [42]. Five studies utilised the list as an intervention, whereas one study [43] used the list as an evaluative element that formed part of a different intervention (clinical medication review).

#### Instrument effects.

Outcome-related data from the identified studies are shown in Tables 7 and 8. Outcomes in all reported studies were assessed by members of the study teams. Three studies reported changes in clinical outcomes following the intervention as change in the number of ADR symptoms at follow-up [37,39,43]. The remaining three studies [41,42,44] only reported ADE identification/documentation rates, with no measurement of change in a condition over time. There were too few data to detect any differences between stand-alone instruments (Table 7) and instruments used as components of interventions (Table 8).

**Table 7:** Study results – ADR identification is the main outcome of the study.

Study first author (date)	Outcome(s) of interest	Measure	Instrument arm	Control arm	Test values, df and P value or 95% confidence interval
**Weingard (2013) [** **44** **]**	Rate of ADEs (primary outcome)	Number of events	98/375 (26.1%)	93/363 (25.6%)	P = 0.89, test values and df not reported. No adjusted analyses reported.
		Preventable	6/375 (1.6%)	2/363 (0.6%)	P = 0.22, test values and df not reported. No adjusted analyses reported.
		Ameliorable	24/375 (6.4%)	19/363 (5.2%)	P = 0.43, test values and df not reported. No adjusted analyses reported.
**Schoenmakers (2017) [39**,**40]**	Drug associated symptoms (primary outcome).	Mean, adjusted for baseline.	Baseline: 5.1 Follow-up: 4.0 Standard deviation (SD) not reported.	Baseline: 4.8 Follow-up: 5.0 SD not reported.	Bivariate comparisons not reported.
	Difference between allocation arms at follow-up (primary outcome).	Unadjusted incident rate ratio between instrument and control arms.			IRR 0.90, 95% confidence interval (95%CI) = 0.62 to 1.33.
	Persisting drug-associated symptoms (reported at baseline and again at follow-up) (secondary outcome).	Mean (percentage of persisting symptoms)	2.1 (43) SD not reported.	2.6 (54) SD not reported.	Bivariate comparisons not reported.
	Persisting symptom difference between allocation arms at follow-up (secondary outcome).	Incident rate ratio			IRR 0.85, 95%CI = 0.43 to 1.42
	Total number of patients reporting ≥1 drug-associated symptom at follow-up (primary outcome).	Number (percentage)	56/78 (72)	51/67 (76)	
	Difference between allocation arms (primary outcome).	Odds ratio ([OR], adjusted for differences at baseline).			aOR 0.85, 95%CI = 0.38 to 1.88
**Schiff (2018) [** **41** **]**	ADE documentation (primary outcome).	Total participants	776	776	
		Total symptoms documented in medical notes.	277	164	P < 0.01 test values and df not reported
		Symptoms per 100 patients.	36	21	P < 0.01 test values and df not reported
		Total unique symptoms documented in interactive voice response calling system and medical notes.	1303	164	P < 0.01 test values and df not reported
	Medicine discontinuation for ADEs (secondary outcome).	Number (percentage)	254 (15)	217 (11)	P = 0.01 test values and df not reported.
		EQ-5D-5L mean (SD) at 6 months	0.73 (0.20)	0.74 (0.18)	
**Buchet-Poyau (2021) [** **42** **]**	Reporting of ADEs (secondary outcome)	Number of ADEs reported (percentage)	69/83 (83.1)	52/65 (80.0)	Not reported
	Patient-reported ADEs (primary outcome)	Number of patients reporting at least one ADE (percentage)	31/546 (5.7)	13/549 (2.4)	
	Impact of intervention on patient self-reporting (n = 908) (primary outcome).	OR (adjusted for the period, age, sex, educational level, morbidity, situations with high risk of ADEs and antihypertensive prescription).			aOR 3.5 95%CI = 1.2 to 10.1
	Association of medicine changes with ADE reporting (secondary outcome).	Adjusted OR (aOR)	Modification		aOR 4.4, 95%CI = 1.9 to 10.0
			Initiation		aOR 11.0, 95%CI = 4.6 to 26.4

Studies are arranged in chronological order.

**Table 8:** Study results – ADR identification is part of a wider intervention.

Study first author (date)	Outcome(s) of interest	Measure	Instrument arm	Control arm	Test values, df and P value or 95% confidence interval
**Jameson (2001) [** **43** **]**	Change in ADE scores at the end of study (6 months), each ADE symptom was rated on a scale from 0 (no trouble) to 3 (a lot of trouble). (primary/secondary outcomes not distinguished by authors).	Improved (≥2 points)	67/124 (54.0%)	58/144 (40.2%)	χ2 overall comparison of groups, P = 0.02, test values and df not reported. No adjusted analyses reported.
		Unchanged (−1 to +1 points)	30/124 (24.2%)	50/144 (34.7%)	
		Worsened (≥2 points)	27/124 (21.8%)	46/144 (31.9%)	
**Verdoorn (2019) [37**,**38]**		Total participants	315	314	
	Difference in the number of health problems at 6 months (primary outcome).	Mean (SD) at baseline (unadjusted).	5.9 (3.0)	5.5 (2.9)	Effect of intervention at 6 months = −0.30, 95%CI = −0.64 to 0.05. P = 0.1 (linear mixed model, accounting for time, intervention and their interaction, age, sex and pharmacy).
		Mean (SD) at 6 months (unadjusted)	5.5 (3.0)	5.3 (2.9)	
	Difference in the number of health problems with impact on daily life at 6 months (primary outcome).	Mean (SD) at baseline (unadjusted).	2.8 (2.4)	2.6 (2.4)	Effect of intervention at 6 months = −0.34, 95%CI = −0.62 to −0.04. P = 0.02 (linear mixed model, accounting for time, intervention and their interaction, age, sex and pharmacy).
		Mean (SD) at 6 months (unadjusted).	2.4 (2.4)	2.5 (2.4)	
	Difference in impact on daily life at 6 months (primary outcome).	EQ-VAS mean score (SD) at baseline.	68 (16)	70 (16)	Effect for intervention at 6 months compared to control = + 3.4 points in quality of life, 95%CI = 0.94 to 5.8. P = 0.01 (linear mixed model, accounting for time, intervention and their interaction, age, sex and pharmacy).
		EQ-VAS mean score (SD) at 6 months.	70 (16)	69 (15)	
		EQ-5D-5L mean (SD) at baseline.	0.73 (0.18)	0.74 (0.18)	Effect for intervention at 6 months compared to control = 0.00, 95%CI = −0.02 to 0.02. P = 0.85 (linear mixed model, accounting for time, intervention and their interaction, age, sex and pharmacy).
		EQ-5D-5L mean (SD) at 6 months.	0.73 (0.20)	0.74 (0.18)	

### Instrument effects in identifying ADRs

One study’s [44] automated electronic message system, which used a generic list of ADRs resulted in a small, statistically insignificant, increase in rate of ADR reporting, compared with a telephone call asking patients about ADRs but not using a list (98 ADEs per 100 patients [26.1%] vs. 93 ADEs per 100 patients [25.6%], p =  0.89, sample size =  738). Use of an interactive voice response automated calling system with live transfer to a pharmacist in another study [41] resulted in a statistically significantly higher number of identified ADRs in the intervention group, compared with propensity-matched controls receiving usual care, as measured by detailed manual chart review (36 symptoms per 100 patients vs. 21 symptoms per 100 patients, p < 0.01, sample size =  1552). In the third study [42], an educational booklet that included a list of ADRs was significantly associated with an increase in the proportion of patients who reported at least one ADR to their general practitioner, when adjusted for the period, age, sex, educational level, morbidity, situations with a risk of ADRs and antihypertensive prescription (aOR =  3.5, 95%CI = 1.2 to 10.1, sample size =  1102). One study (Jameson) calculated costs and found no differences between arms at six months. No studies specified information on the resources and training required to implement the interventions.

### Instrument effects in reducing ADRs

A medicine review or consultation was instrumental (alongside the ADR questionnaire) in the studies that investigated change in the number of ADRs. In one study [43], a medication consultation that included a doctor-pharmacist collaboration resulted in a larger proportion of patients improving and fewer worsening in the instrument arm, compared with the usual care control arm, as measured with an 18-item ADR questionnaire (67 patients [54%] vs. 58 patients [40%], and 27 patients [21.8%] vs. 46 patients [31.9%], p =  0.02, no other test results relating to ADRs are available in the paper, sample size =  340). In another study [39], a pharmacist medicines review (proceeding from a patient-completed ADR questionnaire) reduced the mean number of drug-associated symptoms at follow-up in the instrument arm (by 4.0) compared with the usual care control arm (5.0), but this did not reach statistical significance (incident rate ratio between groups 0.90, 95%CI 0.43 to 1.42, sample size =  228). The third study [37] reported a 12% decrease in the number of problems with impact on daily life in the instrument arm (difference at 6 months −0.34, 95%CI −0.62 to −0.044, sample size =  629), while health-related quality of life increased by 3.4 points (95%CI 0.94 to 5.8) when measured with Euro Quality of Life Visual Analogue Scale. However, no significant difference was detected with the Euro Quality of Life 5D-5L instrument (difference at 6 months =  −0.0022, 95%CI −0.64 to 0.054).

**Fig 3:**
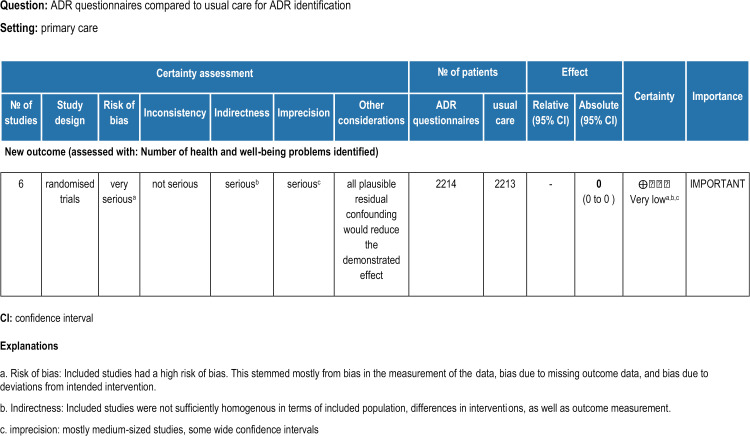
GRADE assessment.

The quality of the evidence and certainty of assessment were facilitated by the Grading of Recommendations, Assessment, Development and Evaluation (GRADE) approach [34,35]. The key assessment components of the approach (see Fig 3) indicated very low certainty of evidence. Included studies had a high risk of bias and the quality of evidence was further downgraded by indirectness [34] stemming from: the heterogeneity of the studied populations (for example in age, number of prescribed medicines, setting); differences in interventions (for example, clinical consultations, patient-reported questionnaires, educational booklet, delivery in person or online or over the telephone, written or spoken communication); and outcome measurement (or example, predefined clinical problems or physician documented problems [Table 6]). Most of the results had wide confidence intervals and moderate sample sizes, contributing to difficulties in drawing reliable conclusions about effects of the intervention due to imprecision and heterogeneity in outcomes. We reviewed the potential for confounding in the trials identified, given the vulnerability of RCTs, particularly small RCTs, to confounding [46].Due to concerns related to the risk of bias, imprecision, and indirectness or variability of outcome measures, the overall certainty and confidence in the evidence is very low.

## Discussion

Although the certainty of evidence for benefit is very low, there is no evidence that the interventions caused harm. All six studies included in this systematic review indicated that ADR detection instruments were safe, and no harms were reported. Of three studies examining effectiveness in ADR identification in adult or older service users, two reported positive results: a booklet, designed to improve patient–GP communication was associated with significant increases of ADR reporting [42], and an automated telephone system implementation resulted in a 50% increase in ADR or ADE symptoms documented in the instrument arm [41]. One study [44], did not find significant impact of the web-based messaging system on the rate of ADEs identified, or healthcare utilisation.

Effects of an instrument at identifying ADRs may be associated with the level of comprehensiveness of the ADR symptom list [47]. While one study [41], demonstrating a positive effect, included 21 items, another study [44], which had no impact, included only 12 ADR symptoms, and it is, therefore, possible that more participants experienced symptoms that were not included in the questionnaire. Increased comprehensiveness of the ADR questionnaire is likely to result in increased effectiveness of the instrument. However, a longer instrument may make more demands on professionals’ time.

Additionally, the lack of statistically significant changes between rates of ADR identification when using a list or not using a list in Weingart’s study [44] may be explained by participant selection bias. Research participation required active use of an electronic health portal, and by extension a presumed relatively high level of IT literacy, which is associated with a high level of health literacy and self-efficacy [48]. Health-literate populations may be expected to be familiar with the most commonly known ADRs (for example by reading online or packet-inserts/patient information leaflets); therefore, utilising instruments listing just a few common symptoms is unlikely to affect this population. The effect of utilising a comprehensive list in populations with high health literacy is unknown. None of the instruments contained comprehensive ADR lists; they included a mean of only 17.8 (SD =  4.87) of the most commonly known ADRs (note: the number of items in Buchet-Poyau’s study [41] is not available). This may suggest that lists, particularly shorter lists, may be most useful with patient populations with low health literacy.

Printed symptom questionnaires followed by medication review with prescribers resulted in no statistically significant reduction in the numbers of drug-associated problems at follow-up [39]. In contrast, symptom questionnaires administered by telephone, followed by a consultation resulted in an ADR score improvement of 2 or more points in the instrument arm, albeit the benefit was smaller than expected [43]. Clinical medication review focusing on personal goals [37] reduced the number of health problems with a moderate or severe impact on daily life at follow-up. Improvement in the quality of life was considerable when measured with a Visual Analogue Scale, but there was no change in health-related quality of life measured with the European Quality of life (EQ-5D-5L) score. It is possible that the improvements experienced were outside the 5 problems (Mobility, Self-care, Usual activities, Pain & discomfort, Anxiety & depression) assessed on the EQ-5D-5L instruments.

Despite variation in primary outcomes, all included studies reported other benefits of their respective interventions. These included: increase in self-efficacy in using medications [39], discontinuations of unnecessary medicines [37,41], participant perception of benefit [43], facilitation of prompt medication-related communication [44] and patient satisfaction with physician and communication [42]. These findings demonstrate the limitations of narrowly defined research or practice outcomes in fully capturing the impact and health and social implications of complex interventions on human subjects [22].

There are few well-designed, low-bias studies evaluating the effectiveness of ADR-identification instruments. The GRADE evaluation therefore indicated limited confidence in any synthesised results. The instruments identified in this review may potentially contribute to solving the problem of how primary care physicians (GPs) find out if their prescribing led to ADRs and how effective it was. ADR lists are convenient, accessible, adaptable and deliverable in different formats. The responses are individualised and reflect the current situation. The comprehensiveness of the lists and the form of delivery affected the number of ADRs identified and the number of people who responded (for example, automated telephone calls had a very poor response rate). Any amelioration of the identified problems involved a prescriber review of the completed ADR list and medicines taken.

Overall, findings of the trials identified reveal the difficulties in detecting effects of complex interventions on patients with complex medication needs in different contexts [49]. Some ADR symptoms may resolve as part of usual care, and some may be transient in nature and resolve without intervention. Some participants may be unwilling to consider changes to their medication regimen, whilst others’ health problems may worsen due to causes unrelated to the intervention. These difficulties were acknowledged in most studies but not resolved.

Examination of interventions that enable effective identification and/or amelioration of ADRs could facilitate prioritisation of primary care processes in medication safety interventions. This is particularly pertinent in the context where common primary care interventions targeting medicine safety and appropriate use of polypharmacy have had limited effects [50–52]. However, current evidence relating to the methods of ADR identification and amelioration is patchy and inconclusive.

Patient – healthcare professional communication appears to be at the core of reducing the burden of ADRs in primary care [26]. It is important to understand the patient perspective, as the type and severity of ADRs can be considered more important than the benefit from the medicine [53]. Individualised approaches reducing inappropriate treatment burden and uncoordinated care are needed to improve treatment outcomes and reduce ADRs [54,55]. Assessment of individual differences among service users and their individual responses to medicines, alongside the complexity and appropriateness of entire medication regimens is crucial [56].

As over a third of older Europeans take 5 or more medications per day [57] and co-morbid complexity and increased number of daily medicines are significant risk factors for ADRs [4,58], people at high risk should receive systematic, proactive and timely surveillance of ADRs to minimise treatment burden [25]. The instruments included in this review could be adapted, integrated and streamlined. However, they lack decision support and required engagement of the highest-paid health care professionals, doctors and pharmacists. Identifying and involving lower-paid healthcare professionals in implementing ADR surveillance strategies within multidisciplinary cooperation, with relevant training, guidance on practical implementation and management support would enable wider use of such ADR identification strategies in clinical practice [59].

### Strengths and limitations of the review

To the authors’ knowledge, this is the first systematic review focussing on identifying and evaluating effects of real-time ADR identification instruments. A combination of MESH terms and commonly used terms was used to search for studies. The reviewers were blinded to one another’s decisions during the study selection process, ensuring rigour.

This systematic review has several possible limitations. The first relates to the strict inclusion criteria, considering only studies published in English and only strategies that have been clinically evaluated in RCTs. The reasons for these restrictions were lack of a translator and the intention to use only the highest level of evidence available [34]. It was not possible to conduct a meta-analysis, due to the heterogeneity of the studies. Additionally, we cannot discount the likely publication bias caused by non-publication of small studies not showing any effect.

The review was limited by suboptimal agreement in terminology relating to polypharmacy, methodology and outcomes in studies evaluating validated ADR identification tools. Uniformity would facilitate future research into identification and appraisal of instruments enabling ADR recognition and amelioration.

Limitations of the data include discordant findings across the different contexts, settings, participants, interventions and outcomes, and paucity of data on clinical or long-term outcomes. It is possible that some of the studies (for example, Weingard et al., [44]) were underpowered to detect a smaller than expected treatment effect. Accordingly, the evidence was downgraded due to high or unclear risk of bias across multiple domains.

Further research is required to establish a robust evidence base for the best methods of identification and amelioration of ADRs in primary care.

## Conclusion

These results suggest that ADR lists probably contribute to ADR identification and using a comprehensive ADR list may well result in more problems being resolved. This systematic review collated empirical evidence of current methods of real-time ADR identification. Currently, it is uncertain whether the findings from this review might support the use of any of the instruments discussed to identify and resolve ADRs. Strategies to detect and/or ameliorate ADRs usually benefit patients and have never been reported as causing harm: accordingly, they warrant further research investment. Comparing participants’ symptoms to a pre-specified list of potential ADRs was a crucial component of all ADR-identification approaches, and combining the ADR lists with prescriber consultation provided the means to mitigate the problems. The methods of delivery of the lists to participants varied, as did processes to manage the identified problems. Some approaches had statistically significant benefits, and all demonstrated participant benefits beyond simple symptom scores. All strategies were reported as safe. Application of comprehensive lists and targeting populations with low health literacy might increase efficiency of the instrument at problem identification, and hence ameliorate the burden of ADRs on patients, hospitals [1,6], and healthcare finances [7,8].

## Supporting information

S1 Fig Stage 2 review inclusion/exclusion decision chart. (PDF)

S2 Fig PRISMA checklist. (DOCX)

S1 File Search strategy and results by database. (DOCX)

S2 File Stage 1 screening. (XLSX)

S3 File Stage 2 screening. (XLSX)

S4 File Tidier description of interventions. (DOCX)

S1 Table Types of studies excluded at stage 2. (DOCX)

S2 Table Studies excluded at stage 3. (DOCX)

## References

[1] World Health Organisanisation. Medication without harm. WHO global patient safety challenge; 2017 [cited 2021 Nov 7]. Available from https://apps.who.int/iris/bitstream/handle/10665/255263/WHO-HIS-SDS-2017.6-eng.pdf?sequence=1

[2] Edwards IR, Aronson JK. Adverse drug reactions: definitions, diagnosis, and management. Lancet. 2000;356(9237):1255–9. doi: 10.1016/S0140-6736(00)02799-9 11072960

[3] Gyllensten H, Rehnberg C, Jönsson AK, Petzold M, Carlsten A, Andersson Sundell K. Cost of illness of patient-reported adverse drug events: a population-based cross-sectional survey. BMJ Open. 2013;3(6):e002574. doi: 10.1136/bmjopen-2013-002574 23794552 PMC3686161

[4] Jennings ELM, Murphy KD, Gallagher P, O’Mahony D. In-hospital adverse drug reactions in older adults; prevalence, presentation and associated drugs-a systematic review and meta-analysis. Age Ageing. 2020;49(6):948–58. doi: 10.1093/ageing/afaa188 33022061

[5] Sakiris MA, Sawan M, Hilmer SN, Awadalla R, Gnjidic D. Prevalence of adverse drug events and adverse drug reactions in hospital among older patients with dementia: a systematic review. Br J Clin Pharmacol. 2021;87(2):375–85. doi: 10.1111/bcp.14417 32520427

[6] Haerdtlein A, Debold E, Rottenkolber M, Boehmer AM, Pudritz YM, Shahid F, et al. Which adverse events and which drugs are implicated in drug-related hospital admissions? A systematic review and meta-analysis. J Clin Med. 2023;12(4):1320. doi: 10.3390/jcm12041320 36835854 PMC9963366

[7] Elliott RA, Camacho E, Jankovic D, Sculpher MJ, Faria R. Economic analysis of the prevalence and clinical and economic burden of medication error in England. BMJ Qual Saf. 2021;30(2):96–105. doi: 10.1136/bmjqs-2019-010206 32527980

[8] Davies J, Cooper RE, Moncrieff J, Montagu L, Rae T, Parhi M. The costs incurred by the NHS in England due to the unnecessary prescribing of dependency-forming medications. Addict Behav. 2022;125:107143. doi: 10.1016/j.addbeh.2021.107143 34674906

[9] Salisbury H. Helen Salisbury: is transactional care enough? BMJ. 2020;368:m226. doi: 10.1136/bmj.m226 31992553

[10] Turner A, Morris R, Rakhra D, Stevenson F, McDonagh L, Hamilton F, et al. Unintended consequences of online consultations: a qualitative study in UK primary care. Br J Gen Pract. 2022;72(715):e128–37. doi: 10.3399/BJGP.2021.0426 34903520 PMC8813120

[11] García-Abeijon P, Costa C, Taracido M, Herdeiro MT, Torre C, Figueiras A. Factors associated with underreporting of adverse drug reactions by health care professionals: a systematic review update. Drug Saf. 2023;46(7):625–36. doi: 10.1007/s40264-023-01302-7 37277678 PMC10279571

[12] Gray SL, Perera S, Soverns T, Hanlon JT. Systematic review and meta-analysis of interventions to reduce adverse drug reactions in older adults: an update. Drugs Aging. 2023;40(11):965–79. doi: 10.1007/s40266-023-01064-y 37702981 PMC10600043

[13] Masnoon N, Shakib S, Kalisch-Ellett L, Caughey GE. What is polypharmacy? A systematic review of definitions. BMC Geriatr. 2017;17(1):230. doi: 10.1186/s12877-017-0621-2 29017448 PMC5635569

[14] Sirois C, Domingues NS, Laroche M-L, Zongo A, Lunghi C, Guénette L, et al. Polypharmacy definitions for multimorbid older adults need stronger foundations to guide research, clinical practice and public health. Pharmacy (Basel). 2019;7(3):126. doi: 10.3390/pharmacy7030126 31470621 PMC6789889

[15] Frazier SC. Health outcomes and polypharmacy in elderly individuals: an integrated literature review. J Gerontol Nurs. 2005;31(9):4–11. doi: 10.3928/0098-9134-20050901-04 16190007

[16] Avery A, Barber N, Ghaleb M, Franklin BD, Armstrong S, Crowe S et al. Investigating the prevalence and causes of prescribing errors in general practice: the PRACtICe study. London: General Medical Council; 2012. Available from: https://www.rpharms.com/Portals/0/Documents/Old%20news%20documents/news%20downloads/gmc-report.pdf

[17] Payne RA, Abel GA, Avery AJ, Mercer SW, Roland MO. Is polypharmacy always hazardous? A retrospective cohort analysis using linked electronic health records from primary and secondary care. Br J Clin Pharmacol. 2014;77(6):1073–82. doi: 10.1111/bcp.12292 24428591 PMC4093932

[18] Prasad N, Lau ECY, Wojt I, Penm J, Dai Z, Tan ECK. Prevalence of and risk factors for drug-related readmissions in older adults: a systematic review and meta-analysis. Drugs Aging. 2024;41(1):1–11. doi: 10.1007/s40266-023-01076-8 37864770 PMC10770220

[19] Guthrie B, Makubate B, Hernandez-Santiago V, Dreischulte T. The rising tide of polypharmacy and drug-drug interactions: population database analysis 1995-2010. BMC Med. 2015;13:74. doi: 10.1186/s12916-015-0322-7 25889849 PMC4417329

[20] Khezrian M, McNeil CJ, Murray AD, Myint PK. An overview of prevalence, determinants and health outcomes of polypharmacy. Ther Adv Drug Saf. 2020;11:2042098620933741. doi: 10.1177/2042098620933741 32587680 PMC7294476

[21] Jordan S, Gabe M, Newson L, Snelgrove S, Panes G, Picek A, et al. Medication monitoring for people with dementia in care homes: the feasibility and clinical impact of nurse-led monitoring. ScientificWorldJournal. 2014;2014:843621. doi: 10.1155/2014/843621 24707218 PMC3951004

[22] Hughes D, Jordan M, Logan PA, Willson A, Snelgrove S, Storey M, et al. Looking for the “Little Things”: a multi-disciplinary approach to medicines monitoring for older people using the ADRe resource. Geriatrics (Basel). 2020;5(4):79. doi: 10.3390/geriatrics5040079 33086499 PMC7709700

[23] Alldred DP, Kennedy M-C, Hughes C, Chen TF, Miller P. Interventions to optimise prescribing for older people in care homes. Cochrane Database Syst Rev. 2016;2(2):CD009095. doi: 10.1002/14651858.CD009095.pub3 26866421 PMC7111425

[24] Jordan S, Tunnicliffe C, Sykes A. Minimizing side-effects: the clinical impact of nurse-administered “side-effect” checklists. J Adv Nurs. 2002;37(2):155–65. doi: 10.1046/j.1365-2648.2002.02064.x 11851783

[25] Jordan S, Logan V, Turner A, Hughes D. Using nurse-led patient monitoring to avoid medicines-related harm. Nurs Stand. 2021;36(7):61–6. doi: 10.7748/ns.2021.e11770 34180162

[26] Lim R, Ellett LK, Roughead EE, Cheah PY, Masnoon N. Patient-reported questionnaires to identify adverse drug reactions: a systematic review. Int J Environ Res Public Health. 2021;18(22):11877. 34831635 10.3390/ijerph182211877 PMC8624083

[27] Atmaja DS, Yulistiani, Suharjono, Zairina E. Detection tools for prediction and identification of adverse drug reactions in older patients: a systematic review and meta-analysis. Sci Rep. 2022;12(1):13189. doi: 10.1038/s41598-022-17410-w 35915219 PMC9341414

[28] Page MJ, McKenzie JE, Bossuyt PM, Boutron I, Hoffmann TC, Mulrow CD, et al. The PRISMA 2020 statement: an updated guideline for reporting systematic reviews. BMJ. 2021;372:n71. doi: 10.1136/bmj.n71 33782057 PMC8005924

[29] Logan V, Jordan S, Hughes D, Turner A. Methods of adverse drug reaction identification in adult service users living in their own homes: a systematic review. PROSPERO; 2022. p. CRD42022322374. Available from: https://www.crd.york.ac.uk/prospero/display_record.php?ID=CRD42022322374

[30] Logan V, Jordan S, Hughes D, Turner A. Methods of adverse drug reaction identification in adult service users living in theirown homes: a systematic review search strategy. Available from: https://www.crd.york.ac.uk/PROSPEROFILES/322374_STRATEGY_20220331.pdf

[31] World Health Organisanisation. Medication safety in polypharmacy. 2019 [cited 2021 Nov 10]. Available from: https://apps.who.int/iris/bitstream/handle/10665/325454/WHO-UHC-SDS-2019.11-eng.pdf?ua=1

[32] Sterne JAC, Savović J, Page MJ, Elbers RG, Blencowe NS, Boutron I, et al. RoB 2: a revised tool for assessing risk of bias in randomised trials. BMJ. 2019;366:l4898. doi: 10.1136/bmj.l4898 31462531

[33] Eldridge S, Campell MK, Drahota AK, Giraudeau B, Reeves BC, Siegfried N, Higgins JPT. Revised Cochrane risk-of-bias tool for cluster-randomized trials (RoB 2 CRT). 2021 [cited 2023 Sept 12]. Available from: https://drive.google.com/file/d/1a7HtfocC74obX1NfW5YnKS0-4pEmis92/view

[34] Guyatt G, Oxman AD, Akl EA, Kunz R, Vist G, Brozek J, et al. GRADE guidelines: 1. Introduction-GRADE evidence profiles and summary of findings tables. J Clin Epidemiol. 2011;64(4):383–94. doi: 10.1016/j.jclinepi.2010.04.026 21195583

[35] Schünemann H, Brożek J, Guyatt T, Oxman A. GRADE handbook. 2013 [cited 2023 Aug 2]. Available from: https://gdt.gradepro.org/app/handbook/handbook.html

[36] Kanellopoulou A, Tsokani S. Handling heterogeneity in Cochrane reviews. 2023 [cited 2023 May 25]. Available from: https://training.cochrane.org/msu-web-clinic-april-2023

[37] Verdoorn S, Blom J, Vogelzang T, Kwint H-F, Gussekloo J, Bouvy ML. The use of goal attainment scaling during clinical medication review in older persons with polypharmacy. Res Social Adm Pharm. 2019;15(10):1259–65. doi: 10.1016/j.sapharm.2018.11.002 30425008

[38] Verdoorn S, Kwint H-F, Blom JW, Gussekloo J, Bouvy ML. Effects of a clinical medication review focused on personal goals, quality of life, and health problems in older persons with polypharmacy: a randomised controlled trial (DREAMeR-study). PLoS Med. 2019;16(5):e1002798. doi: 10.1371/journal.pmed.1002798 31067214 PMC6505828

[39] Schoenmakers TWA, Teichert M, Wensing M, de Smet PAGM. Evaluation of potentially drug-related patient-reported common symptoms assessed during clinical medication reviews: a cross-sectional observational study. Drug Saf. 2017;40(5):419–30. doi: 10.1007/s40264-017-0504-7 28205099 PMC5384965

[40] Schoenmakers TWA, Wensing M, De Smet PAGM, Teichert M. Patient-reported common symptoms as an assessment of interventions in medication reviews: a randomised, controlled trial. Int J Clin Pharm. 2018;40(1):126–34. doi: 10.1007/s11096-017-0575-7 29209863 PMC5840243

[41] Schiff GD, Klinger E, Salazar A, Medoff J, Amato MG, John Orav E, et al. Screening for adverse drug events: a randomized trial of automated calls coupled with phone-based pharmacist counseling. J Gen Intern Med. 2019;34(2):285–92. doi: 10.1007/s11606-018-4672-7 30291602 PMC6374268

[42] Buchet-Poyau K, Chanelière M, Touzet S, Figon S, Rabilloud M, Coli C. Efficiency of an interactive program for enhancing patient reporting of adverse drug events in primary care. Revue d’Épidémiologie et de Santé Publique. 2018;66:S410. doi: 10.1016/j.respe.2018.05.475

[43] Jameson JP, VanNoord GR. Pharmacotherapy consultation on polypharmacy patients in ambulatory care. Ann Pharmacother. 2001;35(7–8):835–40. doi: 10.1345/aph.10259 11485129

[44] Weingart SN, Carbo A, Tess A, Chiappetta L, Tutkus S, Morway L, et al. Using a patient internet portal to prevent adverse drug events: a randomized, controlled trial. J Patient Saf. 2013;9(3):169–75. doi: 10.1097/PTS.0b013e31829e4b95 23965840

[45] McGuinness LA, Higgins JPT. Risk-of-bias VISualization (robvis): An R package and Shiny web app for visualizing risk-of-bias assessments. Res Synth Methods. 2021;12(1):55–61. doi: 10.1002/jrsm.1411 32336025

[46] Deaton A, Cartwright N. Understanding and misunderstanding randomized controlled trials. Soc Sci Med. 2018;210:2–21. doi: 10.1016/j.socscimed.2017.12.005 29331519 PMC6019115

[47] Jordan S, Knight J, Pointon D. Monitoring adverse drug reactions: scales, profiles, and checklists. Int Nurs Rev. 2004;51(4):208–21. doi: 10.1111/j.1466-7657.2004.00251.x 15530161

[48] Lawless J, Toronto CE, Grammatica GL. Health literacy and information literacy: a concept comparison. RSR. 2016;44(2):144–62. doi: 10.1108/rsr-02-2016-0013

[49] Skivington K, Matthews L, Simpson SA, Craig P, Baird J, Blazeby JM, et al. Framework for the development and evaluation of complex interventions: gap analysis, workshop and consultation-informed update. Health Technol Assess. 2021;25(57):1–132. doi: 10.3310/hta25570 34590577 PMC7614019

[50] Avery AJ, Rodgers S, Cantrill JA, Armstrong S, Cresswell K, Eden M, et al. A pharmacist-led information technology intervention for medication errors (PINCER): a multicentre, cluster randomised, controlled trial and cost-effectiveness analysis. Lancet. 2012;379(9823):1310–9. doi: 10.1016/S0140-6736(11)61817-5 22357106 PMC3328846

[51] Rankin A, Cadogan C, Patterson S, Kerse N, Cardwell C, Bradley M. Interventions to improve the appropriate use of polypharmacy for older people. Cochrane Database Syst Rev. 2018;2018(9):CD008165–CD008165. 30175841 10.1002/14651858.CD008165.pub4 PMC6513645

[52] Holland R, Bond C, Alldred DP, Arthur A, Barton G, Birt L, et al. Evaluation of effectiveness and safety of pharmacist independent prescribers in care homes: cluster randomised controlled trial. BMJ. 2023;380:e071883. doi: 10.1136/bmj-2022-071883 36787910 PMC9926330

[53] Caughey GE, Tait K, Vitry AI, Shakib S. Influence of medication risks and benefits on treatment preferences in older patients with multimorbidity. Patient Prefer Adherence. 2017;11:131–40. doi: 10.2147/PPA.S118836 28176925 PMC5268332

[54] World Health Organisation. Technical series on safer primary care: multimorbidity. 2016 [cited 2022 Feb 12]. Available from: https://www.who.int/publications/i/item/9789241511650

[55] NICE Guidance. London: National Institute for Health and Care Excellence (NICE); 2020 [cited 2023 May 22]. Available from: https://www.nice.org.uk/guidance/ng102/evidence/evidence-review-4-signposting-and-referral-to-other-services-and-support-pdf-4909943921

[56] Motter FR, Fritzen JS, Hilmer SN, Paniz ÉV, Paniz VMV. Potentially inappropriate medication in the elderly: a systematic review of validated explicit criteria. Eur J Clin Pharmacol. 2018;74(6):679–700. doi: 10.1007/s00228-018-2446-0 29589066

[57] Midão L, Giardini A, Menditto E, Kardas P, Costa E. Polypharmacy prevalence among older adults based on the survey of health, ageing and retirement in Europe. Arch Gerontol Geriatr. 2018;78:213–20. doi: 10.1016/j.archger.2018.06.018 30015057

[58] Liao P-J, Mao C-T, Chen T-L, Deng S-T, Hsu K-H. Factors associated with adverse drug reaction occurrence and prognosis, and their economic impacts in older inpatients in Taiwan: a nested case-control study. BMJ Open. 2019;9(5):e026771. doi: 10.1136/bmjopen-2018-026771 31079084 PMC6530431

[59] Jordan S. Managing adverse drug reactions: an orphan task. J Adv Nurs. 2002;38(5):437–48. doi: 10.1046/j.1365-2648.2002.02205.x 12028277

