# Supplementary figures and images for: Methods for identifying adverse drug reactions in primary care: A systematic review
Source: PLoS One. 2025 Feb 4;20(2):e0317660. doi: 10.1371/journal.pone.0317660 (PMC11793789; doi:10.1371/journal.pone.0317660)

S1 Figure. Stage 2 review inclusion/exclusion chart.

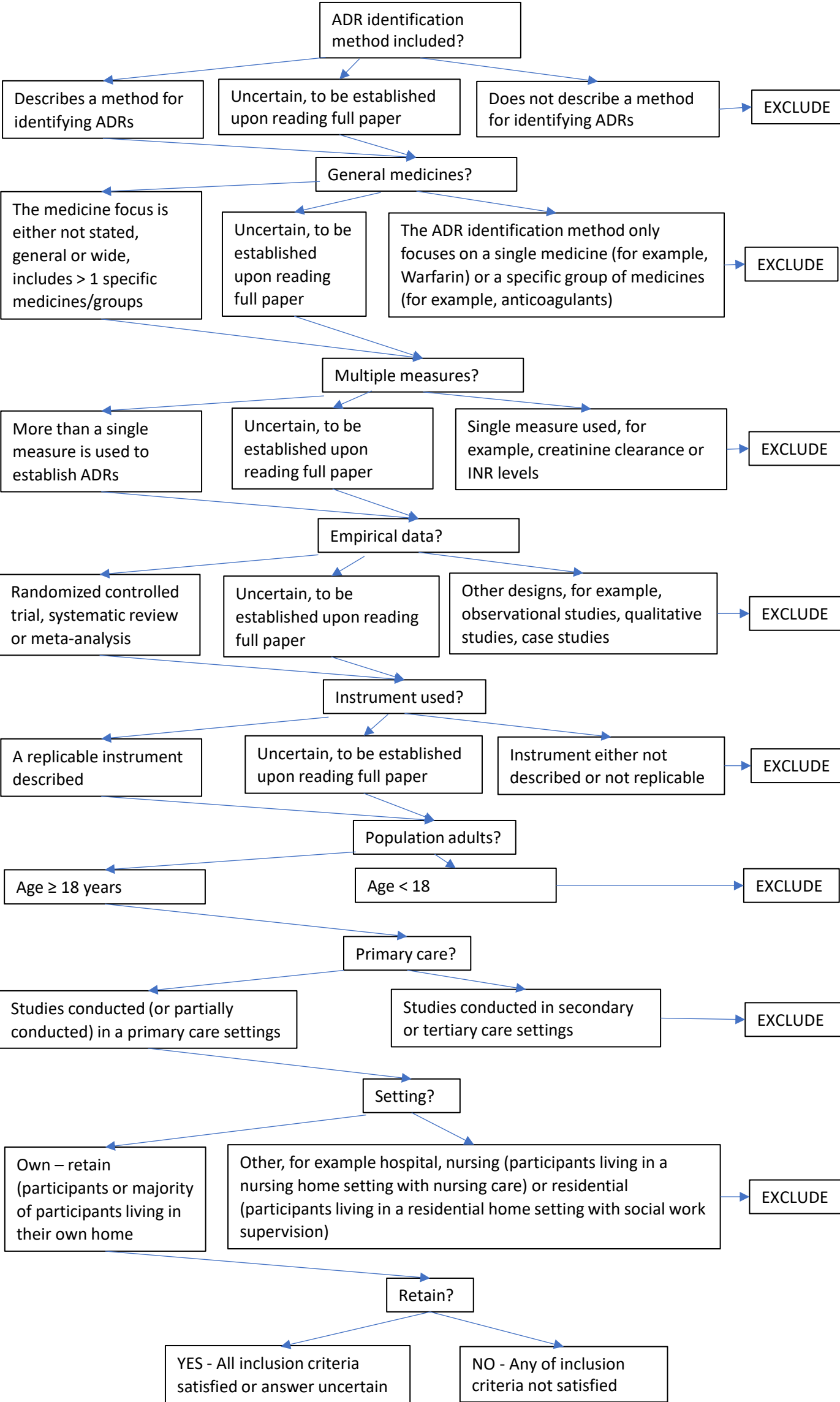

Supplement: S1 Fig — (PDF) [file pone.0317660.s001.pdf]
