# Supplementary material for: Methods for identifying adverse drug reactions in primary care: A systematic review
Source: PLoS One. 2025 Feb 4;20(2):e0317660. doi: 10.1371/journal.pone.0317660 (PMC11793789; doi:10.1371/journal.pone.0317660)
Supplement: S1 File — (DOCX) [file pone.0317660.s003.docx]

**S1 File. Search strategy and results by database.**

**Medline**

| **#** | **Query** | **Limiters/Expanders** | **Last Run Via** | **Results** |
| --- | --- | --- | --- | --- |
| S26 | S18 AND S24 AND S25 | Limiters - Peer Reviewed; Human; Age Related: All Adult: 19+ years; Publication Type: Adaptive Clinical Trial, Clinical Trial, Clinical Trial, Phase I, Clinical Trial, Phase II, Clinical Trial, Phase III, Comparative Study, Controlled Clinical Trial, Equivalence Trial, Meta-Analysis, Pragmatic Clinical Trial, Randomized Controlled Trial, Systematic Review; Language: English Search modes - Find all my search terms | Interface - EBSCOhost Research Databases Search Screen - Advanced Search Database - MEDLINE | 381 |
| S25 | S1 OR S2 OR S3 OR S4 OR S5 OR S6 OR S7 OR S8 OR S9 OR S10 | Expanders - Apply equivalent subjects Search modes - Proximity | Interface - EBSCOhost Research Databases Search Screen - Advanced Search Database - MEDLINE | 208,761 |
| S24 | S19 OR S20 OR S21 OR S22 OR S23 | Expanders - Apply equivalent subjects Search modes - Proximity | Interface - EBSCOhost Research Databases Search Screen - Advanced Search Database - MEDLINE | 1,110,235 |
| S23 | AB ambulatory OR TI ambulatory | Expanders - Apply equivalent subjects Search modes - Proximity | Interface - EBSCOhost Research Databases Search Screen - Advanced Search Database - MEDLINE | 92,155 |
| S22 | AB "family medicine" OR TI "family medicine" OR AB "family practi*" OR TI "family practi*" OR AB "family *care" OR TI "family *care" OR AB "family clinic*" OR TI "family clinic*" | Expanders - Apply equivalent subjects Search modes - Proximity | Interface - EBSCOhost Research Databases Search Screen - Advanced Search Database - MEDLINE | 25,630 |
| S21 | AB "general practi*" OR TI "general practi*" | Expanders - Apply equivalent subjects Search modes - Proximity | Interface - EBSCOhost Research Databases Search Screen - Advanced Search Database - MEDLINE | 93,540 |
| S20 | AB community OR TI community | Expanders - Apply equivalent subjects Search modes - Proximity | Interface - EBSCOhost Research Databases Search Screen - Advanced Search Database - MEDLINE | 796,666 |
| S19 | AB primary N1 *care OR TI primary N1 *care | Expanders - Apply equivalent subjects Search modes - Proximity | Interface - EBSCOhost Research Databases Search Screen - Advanced Search Database - MEDLINE | 175,343 |
| S18 | S11 OR S12 OR S13 OR S14 OR S15 OR S16 OR S17 | Expanders - Apply equivalent subjects Search modes - Proximity | Interface - EBSCOhost Research Databases Search Screen - Advanced Search Database - MEDLINE | 9,571,228 |
| S17 | AB identif* OR TI identif* | Expanders - Apply equivalent subjects Search modes - Proximity | Interface - EBSCOhost Research Databases Search Screen - Advanced Search Database - MEDLINE | 4,437,316 |
| S16 | AB recogni* OR TI recogni* | Expanders - Apply equivalent subjects Search modes - Proximity | Interface - EBSCOhost Research Databases Search Screen - Advanced Search Database - MEDLINE | 959,436 |
| S15 | AB screen* OR TI screen* | Expanders - Apply equivalent subjects Search modes - Proximity | Interface - EBSCOhost Research Databases Search Screen - Advanced Search Database - MEDLINE | 1,044,539 |
| S14 | AB detect* OR TI detect* | Expanders - Apply equivalent subjects Search modes - Proximity | Interface - EBSCOhost Research Databases Search Screen - Advanced Search Database - MEDLINE | 2,947,256 |
| S13 | AB identif* OR TI identif* | Expanders - Apply equivalent subjects Search modes - Proximity | Interface - EBSCOhost Research Databases Search Screen - Advanced Search Database - MEDLINE | 4,437,316 |
| S12 | AB monitor* OR TI monitor* | Expanders - Apply equivalent subjects Search modes - Proximity | Interface - EBSCOhost Research Databases Search Screen - Advanced Search Database - MEDLINE | 1,062,193 |
| S11 | AB strateg* OR TI strateg* | Expanders - Apply equivalent subjects Search modes - Proximity | Interface - EBSCOhost Research Databases Search Screen - Advanced Search Database - MEDLINE | 1,674,853 |
| S10 | AB drug* N3 complication* OR TI drug* N3 complication* | Expanders - Apply equivalent subjects Search modes - Proximity | Interface - EBSCOhost Research Databases Search Screen - Advanced Search Database - MEDLINE | 4,579 |
| S9 | AB drug* N3 "side effect*" OR drug* N3 "side effect*" | Expanders - Apply equivalent subjects Search modes - Proximity | Interface - EBSCOhost Research Databases Search Screen - Advanced Search Database - MEDLINE | 61,606 |
| S8 | AB drug* N3 toxicit* OR TI drug* N3 toxicit* | Expanders - Apply equivalent subjects Search modes - Proximity | Interface - EBSCOhost Research Databases Search Screen - Advanced Search Database - MEDLINE | 23,305 |
| S7 | AB "adverse drug event*" OR TI "adverse drug event*" OR AB "drug adverse event*" OR TI "drug adverse event*" | Expanders - Apply equivalent subjects Search modes - Proximity | Interface - EBSCOhost Research Databases Search Screen - Advanced Search Database - MEDLINE | 5,381 |
| S6 | AB drug* N3 reaction* OR TI drug* N3 reaction* | Expanders - Apply equivalent subjects Search modes - Proximity | Interface - EBSCOhost Research Databases Search Screen - Advanced Search Database - MEDLINE | 33,033 |
| S5 | AB ADR OR TI ADR NOT Adolescent Depression Rating scale NOT Adenoma Detection Rate NOT Adaptive Data Rate | Expanders - Apply equivalent subjects Search modes - Proximity | Interface - EBSCOhost Research Databases Search Screen - Advanced Search Database - MEDLINE | 12,217 |
| S4 | (MM "Adverse Drug Reaction Reporting Systems") OR "adverse drug reaction reporting systems" | Expanders - Apply equivalent subjects Search modes - Proximity | Interface - EBSCOhost Research Databases Search Screen - Advanced Search Database - MEDLINE | 9,378 |
| S3 | ( (MM "Drug Monitoring") OR "drug monitoring" ) OR (MM "Prescription Drug Monitoring Programs") OR "prescription drug monitoring programs" | Expanders - Apply equivalent subjects Search modes - Proximity | Interface - EBSCOhost Research Databases Search Screen - Advanced Search Database - MEDLINE | 37,119 |
| S2 | (MH "Drug-Related Side Effects and Adverse Reactions+/BL/CF/CO/DI/DH/DT/EN/EP/IM /ME/MI/MO/NU/PA/PP/PX/TH/UR/VI/PC") | Expanders - Apply equivalent subjects Search modes - Proximity | Interface - EBSCOhost Research Databases Search Screen - Advanced Search Database - MEDLINE | 65,696 |
| S1 | ( (MM "Drug-Related Side Effects and Adverse Reactions") ) OR ( "Drug-Related Side Effects and Adverse Drug Reactions" ) | Expanders - Apply equivalent subjects Search modes - Proximity | Interface - EBSCOhost Research Databases Search Screen - Advanced Search Database - MEDLINE | 25,672 |

**CINAHL**

| **#** | **Query** | **Limiters/Expanders** | **Last Run Via** | **Results** |
| --- | --- | --- | --- | --- |
| S26 | S18 AND S24 AND S25 | Limiters - English Language; Peer Reviewed; Research Article; Exclude MEDLINE records; Human; Language: English; Publication Type: Clinical Trial, Meta Analysis, Meta Synthesis, Nursing Interventions, Randomized Controlled Trial, Research, Research Instrument, Systematic Review; Age Groups: All Adult Expanders - Apply equivalent subjects Search modes - Find all my search terms | Interface - EBSCOhost Research Databases Search Screen - Advanced Search Database - CINAHL Ultimate | 185 |
| S25 | S1 OR S2 OR S3 OR S4 OR S5 OR S6 OR S7 OR S8 OR S9 OR S10 | Expanders - Apply equivalent subjects Search modes - Proximity | Interface - EBSCOhost Research Databases Search Screen - Advanced Search Database - CINAHL Ultimate | 32,011 |
| S24 | S19 OR S20 OR S21 OR S22 OR S23 | Expanders - Apply equivalent subjects Search modes - Proximity | Interface - EBSCOhost Research Databases Search Screen - Advanced Search Database - CINAHL Ultimate | 455,399 |
| S23 | AB ambulatory OR TI ambulatory | Expanders - Apply equivalent subjects Search modes - Proximity | Interface - EBSCOhost Research Databases Search Screen - Advanced Search Database - CINAHL Ultimate | 28,811 |
| S22 | AB "family medicine" OR TI "family medicine" OR AB "family practi*" OR TI "family practi*" OR AB "family *care" OR TI "family *care" OR AB "family clinic*" OR TI "family clinic*" | Expanders - Apply equivalent subjects Search modes - Proximity | Interface - EBSCOhost Research Databases Search Screen - Advanced Search Database - CINAHL Ultimate | 10,452 |
| S21 | AB "general practi*" OR TI "general practi*" | Expanders - Apply equivalent subjects Search modes - Proximity | Interface - EBSCOhost Research Databases Search Screen - Advanced Search Database - CINAHL Ultimate | 34,010 |
| S20 | AB community OR TI community | Expanders - Apply equivalent subjects Search modes - Proximity | Interface - EBSCOhost Research Databases Search Screen - Advanced Search Database - CINAHL Ultimate | 317,577 |
| S19 | AB primary N1 *care OR TI primary N1 *care | Expanders - Apply equivalent subjects Search modes - Proximity | Interface - EBSCOhost Research Databases Search Screen - Advanced Search Database - CINAHL Ultimate | 100,202 |
| S18 | S11 OR S12 OR S13 OR S14 OR S15 OR S16 OR S17 | Expanders - Apply equivalent subjects Search modes - Proximity | Interface - EBSCOhost Research Databases Search Screen - Advanced Search Database - CINAHL Ultimate | 1,705,189 |
| S17 | AB identif* OR TI identif* | Expanders - Apply equivalent subjects Search modes - Proximity | Interface - EBSCOhost Research Databases Search Screen - Advanced Search Database - CINAHL Ultimate | 880,956 |
| S16 | AB recogni* OR TI recogni* | Expanders - Apply equivalent subjects Search modes - Proximity | Interface - EBSCOhost Research Databases Search Screen - Advanced Search Database - CINAHL Ultimate | 168,402 |
| S15 | AB screen* OR TI screen* | Expanders - Apply equivalent subjects Search modes - Proximity | Interface - EBSCOhost Research Databases Search Screen - Advanced Search Database - CINAHL Ultimate | 233,094 |
| S14 | AB detect* OR TI detect* | Expanders - Apply equivalent subjects Search modes - Proximity | Interface - EBSCOhost Research Databases Search Screen - Advanced Search Database - CINAHL Ultimate | 293,497 |
| S13 | AB identif* OR TI identif* | Expanders - Apply equivalent subjects Search modes - Proximity | Interface - EBSCOhost Research Databases Search Screen - Advanced Search Database - CINAHL Ultimate | 880,956 |
| S12 | AB monitor* OR TI monitor* | Expanders - Apply equivalent subjects Search modes - Proximity | Interface - EBSCOhost Research Databases Search Screen - Advanced Search Database - CINAHL Ultimate | 188,490 |
| S11 | AB strateg* OR TI strateg* | Expanders - Apply equivalent subjects Search modes - Proximity | Interface - EBSCOhost Research Databases Search Screen - Advanced Search Database - CINAHL Ultimate | 375,949 |
| S10 | AB drug* N3 complication* OR TI drug* N3 complication* | Expanders - Apply equivalent subjects Search modes - Proximity | Interface - EBSCOhost Research Databases Search Screen - Advanced Search Database - CINAHL Ultimate | 991 |
| S9 | AB drug* N3 "side effect*" OR drug* N3 "side effect*" | Expanders - Apply equivalent subjects Search modes - Proximity | Interface - EBSCOhost Research Databases Search Screen - Advanced Search Database - CINAHL Ultimate | 3,168 |
| S8 | AB drug* N3 toxicit* OR TI drug* N3 toxicit* | Expanders - Apply equivalent subjects Search modes - Proximity | Interface - EBSCOhost Research Databases Search Screen - Advanced Search Database - CINAHL Ultimate | 2,596 |
| S7 | AB "adverse drug event*" OR TI "adverse drug event*" OR AB "drug adverse event*" OR TI "drug adverse event*" | Expanders - Apply equivalent subjects Search modes - Proximity | Interface - EBSCOhost Research Databases Search Screen - Advanced Search Database - CINAHL Ultimate | 2,554 |
| S6 | AB drug* N3 reaction* OR TI drug* N3 reaction* | Expanders - Apply equivalent subjects Search modes - Proximity | Interface - EBSCOhost Research Databases Search Screen - Advanced Search Database - CINAHL Ultimate | 7,258 |
| S5 | AB ADR OR TI ADR NOT Adolescent Depression Rating scale NOT Adenoma Detection Rate NOT Adaptive Data Rate | Expanders - Apply equivalent subjects Search modes - Proximity | Interface - EBSCOhost Research Databases Search Screen - Advanced Search Database - CINAHL Ultimate | 2,283 |
| S4 | (MM "Adverse Drug Reaction Reporting Systems") OR "adverse drug reaction reporting systems" | Expanders - Apply equivalent subjects Search modes - Proximity | Interface - EBSCOhost Research Databases Search Screen - Advanced Search Database - CINAHL Ultimate | 8,653 |
| S3 | ( (MM "Drug Monitoring") OR "drug monitoring" ) OR (MM "Prescription Drug Monitoring Programs") OR "prescription drug monitoring programs" | Expanders - Apply equivalent subjects Search modes - Proximity | Interface - EBSCOhost Research Databases Search Screen - Advanced Search Database - CINAHL Ultimate | 10,396 |
| S2 | (MH "Drug-Related Side Effects and Adverse Reactions+/BL/CF/CO/DI/DH/DT/EN/EP/IM /ME/MI/MO/NU/PA/PP/PX/TH/UR/VI/PC") | Expanders - Apply equivalent subjects Search modes - Proximity | Interface - EBSCOhost Research Databases Search Screen - Advanced Search Database - CINAHL Ultimate | 156 |
| S1 | ( (MM "Drug-Related Side Effects and Adverse Reactions") ) OR ( "Drug-Related Side Effects and Adverse Drug Reactions" ) | Expanders - Apply equivalent subjects Search modes - Proximity | Interface - EBSCOhost Research Databases Search Screen - Advanced Search Database - CINAHL Ultimate | 27,263 |

**Web of Science**

| Search number | Search Query and Results | Database | Results |
| --- | --- | --- | --- |
| 6 | #1 AND #5 | Web of Science Core Collection Show editionsexpand_more | [796](https://www.webofscience.com/wos/woscc/summary/7050acb8-e51e-44bb-93cb-95dd2461683e-0103d1dc1b/relevance/1) |
| 5 | #2 AND #3 AND #4 and English (Languages) and Review Article or Article (Document Types) | Web of Science Core Collection Show editionsexpand_more | [3,545](https://www.webofscience.com/wos/woscc/summary/3d3b3879-2033-4292-b380-501add9e129b-219b5055/relevance/1) |
| 4 | (((((((((((TS=("Drug-Related Side Effects and Adverse Drug Reactions")) OR TS=("drug monitoring")) OR TS=("prescription drug monitoring programs")) OR TS=("adverse drug reaction reporting systems")) OR TS=(pharmacovigilance)) OR TS=(drug* NEAR/3 reaction*)) OR TS=(ADR NOT Adolescent Depression Rating scale NOT Adenoma Detection Rate NOT Adaptive Data Rate)) OR TS=("adverse drug event*" )) OR TS=("drug adverse event*")) OR TS=(drug* NEAR/3 toxicit* )) OR TS=(drug* NEAR/3 "side effect*")) OR TS=(drug* NEAR/3 complication*) | Web of Science Core Collection Show editionsexpand_more | [128,861](https://www.webofscience.com/wos/woscc/summary/422d4243-a835-4792-bc29-9b6dff1c70e1-219aceb7/relevance/1) |
| 3 | ((((((TS=(strateg*)) OR TS=(monitor*)) OR TS=(identif*)) OR TS=(detect*)) OR TS=(screen*)) OR TS=(recogni*)) OR TS=(identif*) | Web of Science Core Collection Show editionsexpand_more | [17,101,477](https://www.webofscience.com/wos/woscc/summary/eb96fd18-72e6-4eb5-89c2-4d39b2cff159-219aee97/relevance/1) |
| 2 | ((((TS=(primary NEAR/1 *care)) OR TS=(community)) OR TS=("general practi*")) OR TS=("family medicine" OR "family practi*" OR "family *care" OR "family clinic*")) OR TS=(ambulatory) | Web of Science Core Collection Show editionsexpand_more | [2,107,924](https://www.webofscience.com/wos/woscc/summary/5e7f360d-d19f-4665-9618-ae263e341e59-219b0d42/relevance/1) |
| 1 | ((ALL=(trial)) OR ALL=("systematic review")) OR ALL=(metaanalysis) | Web of Science Core Collection Show editionsexpand_more | [2,569,787](https://www.webofscience.com/wos/woscc/summary/3ed0ccb8-2955-4696-adb5-1c9c23fbd8e4-219b90e8/relevance/1) |

**Scopus**

( ( ( TITLE-ABS-KEY ( {Drug-Related Side Effects and Adverse Drug Reactions} ) ) OR ( TITLE-ABS-KEY ( {drug monitoring} OR {prescription drug monitoring programs} ) ) OR ( TITLE-ABS-KEY ( {adverse drug reaction reporting systems} ) ) OR ( TITLE-ABS-KEY ( adr AND not "Adolescent Depression Rating scale" not "Adenoma Detection Rate" not "Adaptive Data Rate" ) ) OR ( TITLE-ABS-KEY ( drug* W/3 reaction* ) ) OR ( TITLE-ABS-KEY ( "drug adverse event" ) ) OR ( TITLE-ABS-KEY ( drug* W/3 toxicit* ) ) OR ( TITLE-ABS-KEY ( drug* W/3 "side effect" ) ) OR ( TITLE-ABS-KEY ( drug* AND n3 AND complication* ) ) ) AND ( ( TITLE-ABS-KEY ( strateg* ) ) OR ( TITLE-ABS-KEY ( monitor* ) ) OR ( TITLE-ABS-KEY ( identif* ) ) OR ( TITLE-ABS-KEY ( detect* ) ) OR ( TITLE-ABS-KEY ( screen* ) ) OR ( TITLE-ABS-KEY ( recogni* ) ) ) AND ( ( TITLE-ABS-KEY ( primary W/1 *care ) ) OR ( TITLE-ABS-KEY ( community ) ) OR ( TITLE-ABS-KEY ( {general practice} ) ) OR ( TITLE-ABS-KEY ( {family medicine} OR {family practice} OR "family care" OR {family clinics} ) ) OR ( TITLE-ABS-KEY ( ambulatory ) ) ) ) AND ( TITLE-ABS-KEY ( trial OR meta-analysis OR {systematic review} ) ) AND LIMIT-TO ( PUBYEAR , 2024 )  OR ( LIMIT-TO ( PUBYEAR , 2023 ) OR LIMIT-TO ( PUBYEAR , 2022 ) OR LIMIT-TO ( PUBYEAR , 2021 ) OR LIMIT-TO ( PUBYEAR , 2020 ) OR LIMIT-TO ( PUBYEAR , 2019 ) OR LIMIT-TO ( PUBYEAR , 2018 ) OR LIMIT-TO ( PUBYEAR , 2017 ) OR LIMIT-TO ( PUBYEAR , 2016 ) OR LIMIT-TO ( PUBYEAR , 2015 ) OR LIMIT-TO ( PUBYEAR , 2014 ) OR LIMIT-TO ( PUBYEAR , 2013 ) OR LIMIT-TO ( PUBYEAR , 2012 ) )

Results: 820
