# Supplementary material for: Methods for identifying adverse drug reactions in primary care: A systematic review
Source: PLoS One. 2025 Feb 4;20(2):e0317660. doi: 10.1371/journal.pone.0317660 (PMC11793789; doi:10.1371/journal.pone.0317660)
Supplement: S4 File — (DOCX) [file pone.0317660.s006.docx]

**S4 File. TIDIER summaries of articles included in the literature review.**

**The TIDieR (Template for Intervention Description and Replication) Checklist*:**

Information to include when describing an intervention and the location of the information.

| **Item number 1** | **Item** | **Where located **** |
| --- | --- | --- |
|  | Jameson 2001 | Primary paper  (Page or appendix  number) |
|  | **BRIEF NAME** |  |
| **1.** | ADR questionnaire and a pharmacotherapy consultation | 836 |
|  | **WHY** |  |
| **2.** | To investigate actual cost and adverse effect outcomes associated with a pharmacotherapy consultation in ambulatory patients receiving polypharmacy. | 836 |
|  | **WHAT** |  |
| **3.** | 18-item questionnaire, tested for internal validity on 50 patients (test/re-test correlation of 0.86).  Questions clustered into 3 domains (cardiovascular symptoms, psychosocial symptoms, drug adverse effects). | 836 |
| **4.** | Procedures:  18-item ADE questionnaire by telephone  Randomisation into consultation group and control group  Consultation:   - Face to face interview (45-60mins), understanding of medications, compliance, disease state control, drug-related problems identified - Pharmacist and clinician meeting to discuss and determine actions - Pharmacist and patient meeting to explain any changes - Repeat 18-item ADE questionnaire | 836 |
|  | **WHO PROVIDED** |  |
| **5.** | Researcher (pharmacist), clinician  All consultations were provided by the researcher (pharmacist) in the office of the patient’s primary care physician | 836 |
|  | **HOW** |  |
| **6.** | Eligible people were contacted by telephone and those willing to participate were given telephone questionnaires to assess adverse drug effect, face to face consultations.  Outcomes:   - Actual medical dnd drug costs - Adverse drug effects and common symptoms - Clinically important improvement or worsening - The numbers of patines improving, staying the same and worsening - Patient feedback about the consultations | 836 |
|  | **WHERE** |  |
| **7.** | Primary care physicians’ offices. 4 large physician practice groups comprising of 19 offices and 133 physicians participated | 836 |
|  | **WHEN and HOW MUCH** |  |
| **8.** | Patients were enrolled from June 1996 to March 1997.  Questionnaire – 2x  Consultation – 1x | 836 |
|  | **TAILORING** |  |
| **9.** | Person-centred intervention, reflecting patient’s problems | 836 |
|  | **MODIFICATIONS** |  |
| **10.^ǂ^** | ? | _____________ |
|  | **HOW WELL** |  |
| **11.** | Planned: fidelity not assessed. | _____________ |
| **12.^ǂ^** | Actual: fidelity not assessed. | _____________ |

** **Authors** - use N/A if an item is not applicable for the intervention being described. **Reviewers** – use ‘?’ if information about the element is not reported/not sufficiently reported.

† If the information is not provided in the primary paper, give details of where this information is available. This may include locations such as a published protocol or other published papers (provide citation details) or a website (provide the URL).

ǂ If completing the TIDieR checklist for a protocol, these items are not relevant to the protocol and cannot be described until the study is complete.

* We strongly recommend using this checklist in conjunction with the TIDieR guide (see *BMJ* 2014;348: g1687) which contains an explanation and elaboration for each item.

* The focus of TIDieR is on reporting details of the intervention elements (and where relevant, comparison elements) of a study. Other elements and methodological features of studies are covered by other reporting statements and checklists and have not been duplicated as part of the TIDieR checklist. When a **randomised trial** is being reported, the TIDieR checklist should be used in conjunction with the CONSORT statement (see [www.consort-statement.org](http://www.consort-statement.org)) as an extension of **Item 5 of the CONSORT 2010 Statement.** When a **clinical trial** **protocol** is being reported, the TIDieR checklist should be used in conjunction with the SPIRIT statement as an extension of **Item 11 of the SPIRIT 2013 Statement** (see [www.spirit-statement.org](http://www.spirit-statement.org)). For alternate study designs, TIDieR can be used in conjunction with the appropriate checklist for that study design (see [www.equator-network.org](http://www.equator-network.org)).

**The TIDieR (Template for Intervention Description and Replication) Checklist*:**

Information to include when describing an intervention and the location of the information

| **Item number 2** | **Item** | **Where located **** |
| --- | --- | --- |
|  | Weingard 2013 | Primary paper  (Page or appendix  number) |
|  | **BRIEF NAME** |  |
| **1.** | Patient internet portal | 169 |
|  | **WHY** |  |
| **2.** | To assess the value of using the patient internet portal enhancing patient-physician communication | 169 |
|  | **WHAT** |  |
| **3.** | Web-based patient portal, electronic messaging system  PatientSite is a web-based patient portal that offers patients the ability to look up their own radiology, pathology and laboratory test results and to request medication renewals, appointments and managed care referrals. It includes an electronic messaging system, enabling communication with providers.  The MedCheck drug safety module works within PatientSite. It automatically queries patients 10 days after they received a new or modified (dose or frequency) prescription. The MedCheck alerts the primary care physician about new prescriptions and provides patients with a list of common midication-related symptoms in addition to a free text symptom field. | 169 |
| **4.** | Procedures:  Electronic enrolment  Randomisation  MedCheck messages when new medication was prescribed or prescription altered (duration: 3 months)  Responses forwarded to the primary care provider  At the end of participants’ enrollment period, a research nurse examined the medical record for the 3 months for evidence of adverse drug events (including drug changes and dose adjustments, reports of medication-related symptoms, urgent visits or referrals, laboratory and radiology test results, demographic information, health care utilisation).  Each participant was offered a telephone interview to discuss any potential adverse drug events. | 170 |
|  | **WHO PROVIDED** |  |
| **5.** | Primary care provider – nurse practitioner, a specialist, internist or other | 170 |
|  | **HOW** |  |
| **6.** | Internet-based, through PatientSite system.  Outcomes:   - Number of adverse drug events (injuries because of medications), preventable adverse drug events and ameliorable adverse drug events. | 170 |
|  | **WHERE** |  |
| **7.** | N/A | _____________ |
|  | **WHEN and HOW MUCH** |  |
| **8.** | 10 days after a patient was prescribed a new medication or when a medication was modified (dose or frequency). | 169 |
|  | **TAILORING** |  |
| **9.** | No tailoring | _____________ |
|  | **MODIFICATIONS** |  |
| **10.^ǂ^** | ? | _____________ |
|  | **HOW WELL** |  |
| **11.** | Planned: | _____________ |
| **12.^ǂ^** | Actual: | _____________ |

** **Authors** - use N/A if an item is not applicable for the intervention being described. **Reviewers** – use ‘?’ if information about the element is not reported/not sufficiently reported.

† If the information is not provided in the primary paper, give details of where this information is available. This may include locations such as a published protocol or other published papers (provide citation details) or a website (provide the URL).

ǂ If completing the TIDieR checklist for a protocol, these items are not relevant to the protocol and cannot be described until the study is complete.

* We strongly recommend using this checklist in conjunction with the TIDieR guide (see *BMJ* 2014;348: g1687) which contains an explanation and elaboration for each item.

* The focus of TIDieR is on reporting details of the intervention elements (and where relevant, comparison elements) of a study. Other elements and methodological features of studies are covered by other reporting statements and checklists and have not been duplicated as part of the TIDieR checklist. When a **randomised trial** is being reported, the TIDieR checklist should be used in conjunction with the CONSORT statement (see [www.consort-statement.org](http://www.consort-statement.org)) as an extension of **Item 5 of the CONSORT 2010 Statement.** When a **clinical trial** **protocol** is being reported, the TIDieR checklist should be used in conjunction with the SPIRIT statement as an extension of **Item 11 of the SPIRIT 2013 Statement** (see [www.spirit-statement.org](http://www.spirit-statement.org)). For alternate study designs, TIDieR can be used in conjunction with the appropriate checklist for that study design (see [www.equator-network.org](http://www.equator-network.org)).

**The TIDieR (Template for Intervention Description and Replication) Checklist*:**

Information to include when describing an intervention and the location of the information

| **Item number 3** | **Item** | **Where located **** |
| --- | --- | --- |
|  | Schoenmakers 2017 | Primary paper  (Page or appendix  number) |
|  | **BRIEF NAME** |  |
| **1.** | Patient-reported outcome measure instrument (PROMISE) | 126 |
|  | **WHY** |  |
| **2.** | To facilitate gathering of meaningful information from patients in clinical medication reviews to detect drug therapy problems | 127 |
|  | **WHAT** |  |
| **3.** | Non-blinded, randomised, controlled trial  Materials: paper-based instrument with 22 pre-defined symptoms of drug therapy problems, self-rated health, concerns and beliefs, self-efficacy relating to medicines, and medication adherence. Patients were asked to report all symptoms experienced in the previous month (yes/no) and to report any suspicion that these symptoms were associated with the drugs they were using (yes/no/don’t know) | 128 |
| **4.** | Procedures:  Patient recruitment by telephone or email  Consent  Randomisation  PROMISE instrument at start of the clinical medication review  Clinical medication review | 128 |
|  | **WHO PROVIDED** |  |
| **5.** | Community pharmacist with access to patients’ medication history in information systems | 128 |
|  | **HOW** |  |
| **6.** | Face to face  Outcomes:  Mean number of drug-associated symptoms  Mean number and types of drug associated symptoms reported at baseline that persisted at follow-up  Number of patients who reported at least one drug-associated symptom at follow-up  A number of days needed to treat persisting drug-associated symptoms  Patient-reported scores on other domains of the PROMISE instrument | 128 |
|  | **WHERE** |  |
| **7.** | Netherlands, primary care/community | _____________ |
|  | **WHEN and HOW MUCH** |  |
| **8.** | Once per participant | 128 |
|  | **TAILORING** |  |
| **9.** | ? | _____________ |
|  | **MODIFICATIONS** |  |
| **10.^ǂ^** | ? | _____________ |
|  | **HOW WELL** |  |
| **11.** | Planned: | _____________ |
| **12.^ǂ^** | Actual: | _____________ |

** **Authors** - use N/A if an item is not applicable for the intervention being described. **Reviewers** – use ‘?’ if information about the element is not reported/not sufficiently reported.

† If the information is not provided in the primary paper, give details of where this information is available. This may include locations such as a published protocol or other published papers (provide citation details) or a website (provide the URL).

ǂ If completing the TIDieR checklist for a protocol, these items are not relevant to the protocol and cannot be described until the study is complete.

* We strongly recommend using this checklist in conjunction with the TIDieR guide (see *BMJ* 2014;348: g1687) which contains an explanation and elaboration for each item.

* The focus of TIDieR is on reporting details of the intervention elements (and where relevant, comparison elements) of a study. Other elements and methodological features of studies are covered by other reporting statements and checklists and have not been duplicated as part of the TIDieR checklist. When a **randomised trial** is being reported, the TIDieR checklist should be used in conjunction with the CONSORT statement (see [www.consort-statement.org](http://www.consort-statement.org)) as an extension of **Item 5 of the CONSORT 2010 Statement.** When a **clinical trial** **protocol** is being reported, the TIDieR checklist should be used in conjunction with the SPIRIT statement as an extension of **Item 11 of the SPIRIT 2013 Statement** (see [www.spirit-statement.org](http://www.spirit-statement.org)). For alternate study designs, TIDieR can be used in conjunction with the appropriate checklist for that study design (see [www.equator-network.org](http://www.equator-network.org)).

**The TIDieR (Template for Intervention Description and Replication) Checklist*:**

Information to include when describing an intervention and the location of the information.

| **Item number4** | **Item** | **Where located **** |
| --- | --- | --- |
|  | Schiff 2018 | Primary paper  (Page or appendix  number) |
|  | **BRIEF NAME** |  |
| **1.** | Automated calls and phone-based pharmacist counselling | 285 |
|  | **WHY** |  |
| **2.** | To timely identify symptoms related to ADEs | 285 |
|  | **WHAT** |  |
| **3.** | Materials:  Interactive voice response technology (automated calls) with a possibility of real-time transfer to a live clinical pharmacist for patients reporting potential drug-related symptoms. | 286 |
| **4.** | Procedures:  clinics identified (26 clinics)  Randomisation (by clinic) – 13 clinics randomised to the intervention group, 13 to control, all adult patients who received a prescription for one of the target medications were contacted.  Patients consented  Interactive voice response call  Telephone transfer to a pharmacist | 287 |
|  | **WHO PROVIDED** |  |
| **5.** | pharmacist | 286 |
|  | **HOW** |  |
| **6.** | Automated telephone call and a live telephone call.  Outcome measures:   - Physician documentation of any adverse effects associated with the target newly prescribed medication (assessed by manual chart review) | 286 |
|  | **WHERE** |  |
| **7.** | The interactive voice response system was deployed to call patients newly prescribed medications for hzpertenseion, diabetes, insomnia and depression | _____________ |
|  | **WHEN and HOW MUCH** |  |
| **8.** | One intervention per participant, the interactive telephone calls occurred one month and four months after receiving a new prescription for one of the inclusion medications. | 287 |
|  | **TAILORING** |  |
| **9.** | Patients with newly prescribed medications for hypertension, diabetes, insomnia and depression. | _____________ |
|  | **MODIFICATIONS** |  |
| **10.^ǂ^** | ? | _____________ |
|  | **HOW WELL** |  |
| **11.** | Planned: | _____________ |
| **12.^ǂ^** | Actual: | _____________ |

** **Authors** - use N/A if an item is not applicable for the intervention being described. **Reviewers** – use ‘?’ if information about the element is not reported/not sufficiently reported.

† If the information is not provided in the primary paper, give details of where this information is available. This may include locations such as a published protocol or other published papers (provide citation details) or a website (provide the URL).

ǂ If completing the TIDieR checklist for a protocol, these items are not relevant to the protocol and cannot be described until the study is complete.

* We strongly recommend using this checklist in conjunction with the TIDieR guide (see *BMJ* 2014;348: g1687) which contains an explanation and elaboration for each item.

* The focus of TIDieR is on reporting details of the intervention elements (and where relevant, comparison elements) of a study. Other elements and methodological features of studies are covered by other reporting statements and checklists and have not been duplicated as part of the TIDieR checklist. When a **randomised trial** is being reported, the TIDieR checklist should be used in conjunction with the CONSORT statement (see [www.consort-statement.org](http://www.consort-statement.org)) as an extension of **Item 5 of the CONSORT 2010 Statement.** When a **clinical trial** **protocol** is being reported, the TIDieR checklist should be used in conjunction with the SPIRIT statement as an extension of **Item 11 of the SPIRIT 2013 Statement** (see [www.spirit-statement.org](http://www.spirit-statement.org)). For alternate study designs, TIDieR can be used in conjunction with the appropriate checklist for that study design (see [www.equator-network.org](http://www.equator-network.org)).

**The TIDieR (Template for Intervention Description and Replication) Checklist*:**

Information to include when describing an intervention and the location of the information.

| **Item number 5** | **Item** | **Where located **** |
| --- | --- | --- |
|  | Verdoorn 2018 | Primary paper  (Page or appendix  number) |
|  | **BRIEF NAME** |  |
| **1.** | Personal goals medication review (DREAMeR) | 1 |
|  | **WHY** |  |
| **2.** | To investigate the effect of person-centred medication review focussed on personal goals on health-related quality of life and number of health problems. | 3-4 |
|  | **WHAT** |  |
| **3.** | Materials: self-developed written questionnaire | 4 |
| **4.** | Procedures:  Recruitment  Randomisation (block), the control group patients received usual care, the intervention group receved the following:   - Patient completed an ADE questionnaire - Clinical medication review (discussion of health problems, preferences, medications, problems summarised, health-related goals summarised) - Pharmacist recommendations to solve drug-related problems - Pharmacist – GP face to face meeting to propose a pharmaceutical care plan. - Pharmaceutical care plan discussed with patient - 2 follow-up appointments within 3 months to evaluate agreed actions and attainment of goals | 5 |
|  | **WHO PROVIDED** |  |
| **5.** | Pharmacists (received 1 day training, attended web conferences, and presented one case study)  GPs collaborated | 4-5 |
|  | **HOW** |  |
| **6.** | Face to face, patients in the intervention group received a clinical medication review focused on personal goals. At the end of the interview, the problems were summarised, and health-related goals were proposed. A pharmaceutical care plan was then proposed, agreed and implemented.  Outcome measures:   - Health-related quality of life - Number of health problems | 4 |
|  | **WHERE** |  |
| **7.** | Community pharmacies in the Netherlands, general practices | 4 |
|  | **WHEN and HOW MUCH** |  |
| **8.** | April 2016 – February 2017  Participants followed for 6 months | 5 |
|  | **TAILORING** |  |
| **9.** | Study participants aged >70 years and using >7 long term medications | _____________ |
|  | **MODIFICATIONS** |  |
| **10.^ǂ^** | ? | _____________ |
|  | **HOW WELL** |  |
| **11.** | Planned: | _____________ |
| **12.^ǂ^** | Actual: | _____________ |

** **Authors** - use N/A if an item is not applicable for the intervention being described. **Reviewers** – use ‘?’ if information about the element is not reported/not sufficiently reported.

† If the information is not provided in the primary paper, give details of where this information is available. This may include locations such as a published protocol or other published papers (provide citation details) or a website (provide the URL).

ǂ If completing the TIDieR checklist for a protocol, these items are not relevant to the protocol and cannot be described until the study is complete.

* We strongly recommend using this checklist in conjunction with the TIDieR guide (see *BMJ* 2014;348: g1687) which contains an explanation and elaboration for each item.

* The focus of TIDieR is on reporting details of the intervention elements (and where relevant, comparison elements) of a study. Other elements and methodological features of studies are covered by other reporting statements and checklists and have not been duplicated as part of the TIDieR checklist. When a **randomised trial** is being reported, the TIDieR checklist should be used in conjunction with the CONSORT statement (see [www.consort-statement.org](http://www.consort-statement.org)) as an extension of **Item 5 of the CONSORT 2010 Statement.** When a **clinical trial** **protocol** is being reported, the TIDieR checklist should be used in conjunction with the SPIRIT statement as an extension of **Item 11 of the SPIRIT 2013 Statement** (see [www.spirit-statement.org](http://www.spirit-statement.org)). For alternate study designs, TIDieR can be used in conjunction with the appropriate checklist for that study design (see [www.equator-network.org](http://www.equator-network.org)).

**The TIDieR (Template for Intervention Description and Replication) Checklist*:**

Information to include when describing an intervention and the location of the information

| **Item number 6** | **Item** | **Where located **** |
| --- | --- | --- |
|  | Buchet-Poyau 2021 | Primary paper  (Page or appendix  number) |
|  | **BRIEF NAME** |  |
| **1.** | Booklet designed to improve communication between GPs and patients | 1 |
|  | **WHY** |  |
| **2.** | To assess the impact of the booklet on patient self-reporting of ADEs to their GP | 2 |
|  | **WHAT** |  |
| **3.** | Materials: Paper information booklet delivered by GP (– a cross-sectional stepped wedge cluster randomised trial). The booklet was developed following a 10-step process (including a literature review, 3 rounds of Delphi survey, independent review, readability assessment, observational study, linguistic work and validation of the final version). It included information on cardiovascular risks and management of antihypertensive treatments, care plans, and adverse drug event reporting forms that could be annotated by the patient. | 2 |
| **4.** | Procedures:  Participant identification  Randomisation (stepped wedge cluster)  GP-completed online form (morbidity, drugs, risk of ADEs)  Telephone survey about ADEs (research assistant)  GP delivers booklet during consultation  Research assistant telephone call about ADEs  GP-completed online form – data on ADEs | 3-4 |
|  | **WHO PROVIDED** |  |
| **5.** | 88 GPs grouped into 8 clusters based on geographic proximity.  GP took part in a web seminar on how to deliver the booklet  Research assistant completed telephone call surveys about ADEs | 4 |
|  | **HOW** |  |
| **6.** | Paper booklet was delivered during GP consultation.  Outcomes:   - Reporting of at least one adverse drug event by the patient during the 3-month period after enrollement - Reporting of at least one adverse drug event by the patient or the GP | 4 |
|  | **WHERE** |  |
| **7.** | French GP practices | 4 |
|  | **WHEN and HOW MUCH** |  |
| **8.** | June 2012 – September 2013  5 periods of 3 months (during 1^st^ period, all clusters belonged to control group. After that, the intervention was rolled out sequentially 2 clusters at a time at a random order).  One booklet delivered per participant | 4 |
|  | **TAILORING** |  |
| **9.** | ? | _____________ |
|  | **MODIFICATIONS** |  |
| **10.^ǂ^** | ? | _____________ |
|  | **HOW WELL** |  |
| **11.** | Planned: | _____________ |
| **12.^ǂ^** | Actual: | _____________ |

** **Authors** - use N/A if an item is not applicable for the intervention being described. **Reviewers** – use ‘?’ if information about the element is not reported/not sufficiently reported.

† If the information is not provided in the primary paper, give details of where this information is available. This may include locations such as a published protocol or other published papers (provide citation details) or a website (provide the URL).

ǂ If completing the TIDieR checklist for a protocol, these items are not relevant to the protocol and cannot be described until the study is complete.

* We strongly recommend using this checklist in conjunction with the TIDieR guide (see *BMJ* 2014;348: g1687) which contains an explanation and elaboration for each item.

* The focus of TIDieR is on reporting details of the intervention elements (and where relevant, comparison elements) of a study. Other elements and methodological features of studies are covered by other reporting statements and checklists and have not been duplicated as part of the TIDieR checklist. When a **randomised trial** is being reported, the TIDieR checklist should be used in conjunction with the CONSORT statement (see [www.consort-statement.org](http://www.consort-statement.org)) as an extension of **Item 5 of the CONSORT 2010 Statement.** When a **clinical trial** **protocol** is being reported, the TIDieR checklist should be used in conjunction with the SPIRIT statement as an extension of **Item 11 of the SPIRIT 2013 Statement** (see [www.spirit-statement.org](http://www.spirit-statement.org)). For alternate study designs, TIDieR can be used in conjunction with the appropriate checklist for that study design (see [www.equator-network.org](http://www.equator-network.org)).
