# Supplementary material for: Methods for identifying adverse drug reactions in primary care: A systematic review
Source: PLoS One. 2025 Feb 4;20(2):e0317660. doi: 10.1371/journal.pone.0317660 (PMC11793789; doi:10.1371/journal.pone.0317660)
Supplement: S1 Table — (DOCX) [file pone.0317660.s007.docx]

**Supplementary table 1. Details of studies excluded at stage 2 screening.**

| **Excluded category (number of excluded studies)** | **No ADR identification (174)** | **General applicability /Multiple measures (91)** | **General medicines (61)** | **Other (46)** |
| --- | --- | --- | --- | --- |
| **Sub-category 1**  **(number of excluded studies)** | No ADR identification (167) | Laboratory monitoring only (36) | Diabetic medicines only (16) | No instrument used (23) |
| **Sub-category 2**  **(number of excluded studies)** | Potentially inappropriate medicines identification (7) | INR monitoring only (24) | Oncological medicines only (12) | Not primary care setting (12) |
| **Sub-category 3**  **(number of excluded studies)** |  | Blood pressure monitoring only (15) | Anticoagulants only (10) | Nursing or residential home setting (4) |
| **Sub-category 4**  **(number of excluded studies)** |  | Blood glucose only (9) | Opioids only (9) | No empirical data (6) |
| **Sub-category 5**  **(number of excluded studies)** |  | Heparin monitoring only (4) | Clozapine only (5) | Population not adults (1) |
| **Sub-category 6**  **(number of excluded studies)** |  | Lithium levels only (2) | Digoxin only (4) |  |
| **Sub-category 7**  **(number of excluded studies)** |  | Falls only (1) | Amiodarone only (2) |  |
| **Sub-category 8**  **(number of excluded studies)** |  |  | Levodopa only (1), Felodipine only (1), Fluoropirimidine only (1) |  |

Table note: Studies may have been excluded for more than one reason, only the first reason is recorded in this table.
