# Supplementary material for: Methods for identifying adverse drug reactions in primary care: A systematic review
Source: PLoS One. 2025 Feb 4;20(2):e0317660. doi: 10.1371/journal.pone.0317660 (PMC11793789; doi:10.1371/journal.pone.0317660)
Supplement: S2 Table — (DOCX) [file pone.0317660.s008.docx]

**Supplementary table 2. Studies excluded at stage 3.**

| **Reason for exclusion (number of studies)** | **Studies** |
| --- | --- |
| **No acceptable ADR identification instrument present** | 1. Bomboy KT, Graber JS, Wallis EP. Improved Prescriber Adherence to Guidelines on Antipsychotic Medication Management Through Increased Access to Metabolic Monitoring Forms. Journal of the American Psychiatric Nurses Association. 2021;27(2):162–8.  2. Campins L, Serra-Prat M, Gozalo I, Lopez D, Palomera E, Agusti C, et al. Randomized controlled trial of an intervention to improve drug appropriateness in community-dwelling polymedicated elderly people. Family practice. 2017;34(1):36–42.  3. Clyne B, Smith SM, Hughes CM, Boland F, Bradley MC, Cooper JA, et al. Effectiveness of a Multifaceted Intervention for Potentially Inappropriate Prescribing in Older Patients in Primary Care: A Cluster-Randomized Controlled Trial (OPTI-SCRIPT Study). Annals of family medicine. 2015;13(6):545–53.    4. Dalton K, O’Mahony D, O’Sullivan D, O’Connor MN, Byrne S. Prescriber Implementation of STOPP/START Recommendations for Hospitalised Older Adults: A Comparison of a Pharmacist Approach and a Physician Approach. Drugs & aging. 2019;36(3):279–88.    5. Elliott RA, Martinac G, Campbell S, Thorn J, Woodward MC. Pharmacist-Led Medication Review to Identify Medication-Related Problems in Older People Referred to an Aged Care Assessment Team: A Randomized Comparative Study. Drugs & aging. 2012;29(7):593–605.    6. Kwint HF, Faber A, Gussekloo J, Bouvy ML. Completeness of medication reviews provided by community pharmacists. Journal of clinical pharmacy and therapeutics. 2014;39(3):248–52.    7. Leendertse AJ, De Koning FHP, Goudswaard AN, Jonkhoff AR, Van Den Bogert SCA, De Gier HJ, et al. Preventing hospital admissions by reviewing medication (PHARM) in primary care: Design of the cluster randomised, controlled, multi-centre PHARM-study. BMC health services research. 2011;11(1):4–4.    8. Lim WY, Hss AS, Ng LM, John Jasudass SR, Sararaks S, Vengadasalam P, et al. The impact of a prescription review and prescriber feedback system on prescribing practices in primary care clinics: A cluster randomised trial. BMC family practice. 2018;19(1):120–120.  9. Mason, J. D., & Colley, C. A. Effectiveness of an ambulatory care clinical pharmacist: a controlled trial. The Annals of pharmacotherapy, 1993; 27(5), 555–559.  10. Pammett RT, Blackburn D, Taylor J, Mansell K, Kwan D, Papoushek C, et al. Evaluation of a Community Pharmacy-Based Screening Questionnaire to Identify Patients at Risk for Drug Therapy Problems. Pharmacotherapy. 2015;35(9):881–6.    11. Pit SW, Byles JE, Cockburn J. Medication Review: Patient Selection and General Practitioner’s Report of Drug-Related Problems and Actions Taken in Elderly Australians. Journal of the American Geriatrics Society (JAGS). 2007;55(6):927–34.  12. Ridge A, Macintyre K, Kitsos A, Murray L, Bereznicki L. Assessing risk of adverse drug reactions in the elderly: a feasibility study. International journal of clinical pharmacy. 2019;41(6):1483–90.    13. Riordan DO, Walsh KA, Galvin R, Sinnott C, Kearney PM, Byrne S. The effect of pharmacist-led interventions in optimising prescribing in older adults in primary care: A systematic review. SAGE Open Medicine. 2016;4:2050312116652568–2050312116652568.    14. Singh R, Anderson D, McLean-Plunkett E, Brooks R, Wisniewski A, Satchidanand N, et al. IT-enabled systems engineering approach to monitoring and reducing ADEs. The American journal of managed care. 2012;18(3):169–75.    15.Touchette, D. R., Masica, A. L., Dolor, R. J., Schumock, G. T., Choi, Y. K., Kim, Y., & Smith, S. R. Safety-focused medication therapy management: a randomized controlled trial. Journal of the American Pharmacists Association: JAPhA, 2012;52(5), 603–612.    16. Vinks THAM, Egberts TCG, de Lange TM, de Koning FHP. Pharmacist-Based Medication Review Reduces Potential Drug-Related Problems in the Elderly: The SMOG Controlled Trial. Drugs & aging. 2009;26(2):123–33. |
| **Not accepted methodology** | 1. de Almeida SM, Romualdo A, de Abreu Ferraresi A, Zelezoglo GR, Marra AR, Edmond MB. Use of a trigger tool to detect adverse drug reactions in an emergency department. BMC pharmacology & toxicology. 2017;18(1):71–71.    2. Hirsch BR, Harrison MR, George DJ, Walker MS, Chen C, Korytowsky B, et al. Use of “Real-World” data to describe adverse events during the treatment of metastatic renal cell carcinoma in routine clinical practice. Medical oncology (Northwood, London, England). 2014;31(9):1–8.    3. Judson TJ, Bennett AV, Rogak LJ, Sit L, Barz A, Kris MG, et al. Feasibility of Long-Term Patient Self-Reporting of Toxicities From Home via the Internet During Routine Chemotherapy. Journal of clinical oncology. 2013;31(20):2580–5.    4. Makowsky MJ, Cor K, Wong T. Exploring electronic medical record and self-administered medication risk screening tools in a primary care clinic. Journal of managed care & specialty pharmacy. 2017;23(5):566–72.    5. Masino AJ, Forsyth D, Fiks AG. Detecting Adverse Drug Reactions on Twitter with Convolutional Neural Networks and Word Embedding Features. Journal of healthcare informatics research. 2018;2(1–2):25–43.  6. Pammett RT, Blackburn D, Taylor J, Mansell K, Kwan D, Papoushek C, et al. Evaluation of a Community Pharmacy-Based Screening Questionnaire to Identify Patients at Risk for Drug Therapy Problems. Pharmacotherapy. 2015;35(9):881–6.    7. Shoji M, Maeda H, Watanabe F, Tanuma K, Fujiwara A, Iwanaga Y, et al. A non-randomized, controlled, interventional study to investigate the effects of community pharmacists’ cognitive behavioral therapy–based interventions on medication adherence and relevant indicators in patients with depression. BMC psychiatry. 2023;23(1):124–124.  8. Steinman MA, Handler SM, Gurwitz JH, Schiff GD, Covinsky KE. Beyond the Prescription: Medication Monitoring and Adverse Drug Events in Older Adults. Journal of the American Geriatrics Society (JAGS). 2011;59(8):1513–20.    9. Tam KWT, Kwok HK, Fan YMC, Tsui KB, Ng KK, Ho KYA, et al. Detection and prevention of medication misadventures in general practice. International journal for quality in health care. 2008;20(3):192–9.    10. Westerlund T, Marklund B. Assessment of the clinical and economic outcomes of pharmacy interventions in drug-related problems. Journal of clinical pharmacy and therapeutics. Received 4 June 2008, Accepted 16 October 2008. 2009;34(3):319–27. |
| **Not general, intervention specific to a narrow range or measures** | 1. Ahmed NO, Osman B, Abdelhai YM, El-Hadiyah TMH. Impact of clinical pharmacist intervention in anticoagulation clinic in Sudan. International journal of clinical pharmacy. 2017;39(4):769–73.    2. Farmer AJ, McSharry J, Rowbotham S, McGowan L, Ricci-Cabello I, French DP. Effects of interventions promoting monitoring of medication use and brief messaging on medication adherence for people with Type 2 diabetes: a systematic review of randomized trials. Diabetic medicine. Diabet. Med. 33, 565-579 (2016). 2016;33(5):565–79.    3. Kuipers E, Wensing M, de Smet P, Teichert M. Self-management research of asthma and good drug use (SMARAGD study): a pilot trial. International journal of clinical pharmacy. 2017;39(4):888–96.  4. Leake Date HA, Alford K, Hounsome N, Moore D, Ing K, Vera JH. Structured medicines reviews in HIV outpatients: a feasibility study (The MOR Study). HIV medicine. 2022;23(1):39–47.    5. Palen TE, Raebel M, Lyons E, Magid DM. Evaluation of laboratory monitoring alerts within a computerized physician order entry system for medication orders. The American journal of managed care. 2006;12(7):389–95.    6. Rasmussen LM, Phanareth K, Nolte H, Backer V. Internet-based monitoring of asthma: A long-term, randomized clinical study of 300 asthmatic subjects. Journal of allergy and clinical immunology. 2005;115(6):1137–42.  7. Tjia J, Fischer SH, Raebel MA, Peterson D, Zhao Y, Gagne SJ, et al. Baseline and Follow-up Laboratory Monitoring of Cardiovascular Medications. The Annals of pharmacotherapy. 2011;45(9):1077–84.  8. Twigg G, David T, Taylor J. An Improved Comprehensive Medication Review Process to Assess Healthcare Outcomes in a Rural Independent Community Pharmacy. Pharmacy. 2019;7(2):66-.    9. van Grootheest AC, Groote JK, de Jong-van den Berg LTW. Intensive monitoring of new drugs based on first prescription signals from pharmacists: a pilot study. Pharmacoepidemiology and drug safety. 2003;12(6):475–81. |
| **Oncology medicines focus only** | 1. Moloney M, Faulkner D, Link E, Rischin D, Solomon B, Lim AM, et al. Feasibility of 5-fluorouracil pharmacokinetic monitoring using the My-5FU PCMTM system in a quaternary oncology centre. Cancer chemotherapy and pharmacology. 2018;82(5):865–76.    2. Nikfarjam A, Ransohoff JD, Callahan A, Jones E, Loew B, Kwong BY, et al. Early detection of adverse drug reactions in social health networks: A natural language processing pipeline for signal detection. JMIR public health and surveillance. 2019;21(6):e11264–e11264.    3. Rasschaert M, Vulsteke C, De Keersmaeker S, Vandenborne K, Dias S, Verschaeve V, et al. AMTRA: a multicentered experience of a web-based monitoring and tailored toxicity management system for cancer patients. Supportive care in cancer. 2021;29(2):859–67.    4. Traeger L, McDonnell TM, McCarty CE, Greer JA, El‐Jawahri A, Temel JS. Nursing intervention to enhance outpatient chemotherapy symptom management: Patient‐reported outcomes of a randomized controlled trial. Cancer. 2015;121(21):3905–13. |
| **Ineligible setting** | 1. Gabe ME, Murphy F, Davies GA, Russell IT, Jordan S. Medication monitoring in a nurse-led respiratory outpatient clinic: Pragmatic randomised trial of the west wales adverse drug reaction profile. PloS one. 2014;9(5):e96682–e96682.    2. Jordan S. Managing adverse drug reactions: an orphan task. Journal of advanced nursing. 2002;38(5):437–48. |
